# Neural Mechanisms of Self-Generated Action Sequences

**DOI:** 10.1523/ENEURO.0316-25.2026

**Published:** 2026-05-22

**Authors:** Silvia Seghezzi, Patrick Haggard

**Affiliations:** ^1^University College London, London WC1 3AZ, United Kingdom; ^2^Birkbeck, University of London, London WC1E 7HX, United Kingdom

**Keywords:** EEG, executive functions, problem-solving, readiness potential, volition

## Abstract

Complex problems often allow multiple paths to a solution. Choosing and taking the best path is an important part of the executive cognition that underpins intelligent problem-solving behavior. However, once a path is chosen, the motor system must be activated for executing it. This interface between problem-solving and self-generated action has rarely been studied. We recorded EEG movement-related potentials while 25 participants (7 males, 18 females) performed the “Tower of London” problem-solving task. In a control condition, participants merely followed instructed steps without planning for any goal and thus without any sense that their movements solved a problem. Readiness potentials (RPs) preceding actions showed a more sustained preparatory negativity for self-generated than stimulus-driven movements. Critically, this effect was most pronounced at the first move of a sequence and diminished at later stages, indicating that preparatory activity is closely linked to the planning demands of sequence initiation. Consistent with this, contralateral motor β-band suppression was stronger for self-generated actions, particularly at sequence onset, but remained present across all moves, indicating that it is not selectively modulated by sequence position in the same way as the RP. Multivariate pattern analysis further showed that self-generated and stimulus-driven actions could be reliably distinguished throughout the entire preparatory period. Taken together, these results show a deep interaction between executive function and self-generated actions and draw attention to the fact that, if a problem can be solved, then actually solving it generally requires executive cognition to trigger self-generated actions, based on a plan.

## Significance Statement

Self-generated actions are often studied as isolated, arbitrary movements, whereas real behavior typically involves solving problems through sequences of goal-directed acts. Using EEG during a modified Tower of London task, we show that neural markers of self-generated action are strongest when participants initiate a planned action sequence and diminish as the sequence unfolds. Readiness potentials, motor beta suppression, and multivariate decoding all distinguished self-generated from stimulus-driven actions during preparation. These results show that the neural signatures of self-generated action are closely linked to planning demands, bridging the neuroscience of volition with the psychology of problem-solving.

## Introduction

People solve their problems by doing things. Despite this rather evident truth, scientific research on problem-solving and on self-generated action has proceeded largely independently. Research on problem-solving has largely followed the neuropsychological traditions of executive function (Cooper and Shallice, 2000; Gilbert and Burgess, 2008), focusing on aspects such as conflict resolution, optimal choices, and hierarchical representation of sequences (Koechlin et al., 2000, 2003; Koechlin and Hyafil, 2007; Koechlin and Summerfield, 2007; Botvinick, 2008; Botvinick and Cohen, 2014). However, real-world problems often go beyond searches for an optimal path, since they may involve generating and committing to one of several possible strategies that can all achieve the goal without differences in cost.

Conversely, neuroscientific studies of self-generated action have rarely considered the relations between processes of self-generated action generation and optimality or complexity of behavior. Instead, they have focused on the question of whether and how mental processes of self-generated actions could provide an initial cause for neural precursors for action (Libet et al., 1983). This program has been criticized on metaphysical grounds as being too dualistic (Dennett and Kinsbourne, 1992). Here we instead focus on a second, separate concern: the existing neuroscience of self-generated actions has largely neglected the obvious compositionality and teleology of self-generated action. Many self-generated actions occur as just one part in a complex sequence of behavior that aims toward a distal goal with meaningful and transformative effects. For example, many experimental studies, including ours, focus on simple button-press movements. However, outside the experimental laboratory, the same action would form part of a complex, purposive sequence. One might press the button to switch on the light, so one can read the book.

The core finding in the cognitive neuroscience of self-generated actions is perhaps the consistent pattern of EEG activity found prior to self-paced actions. Kornhuber and Deecke (1965) were the first to report a gradual ramp-like increase in premovement EEG negativity, which they termed readiness potential (RP; Kornhuber and Deecke, 1965). Several studies have attempted to estimate the onset time of this signal. However, these attempts often depend on specifying baseline-correction periods for EEG, which may reflect arbitrary and unjustified assumptions about when precursor activity begins (Khalighinejad et al., 2019). Recent computational models suggest that the search for a specific event that initiates the RP is, in fact, a misguided category mistake (Schurger et al., 2012).

Interestingly, a further reliable marker of impending action is the reduction in beta-band (13–30 Hz, beta-ERD, beta event-related desynchronization) EEG power during motor preparation (Stancák and Pfurtscheller, 1996; Pfurtscheller and Lopes da Silva, 1999). This drops progressively over time, typically reaching a minimum contralateral to the action, at the time movement is initiated (O'Connell et al., 2012; Steinemann et al., 2018). While the RP build up and motor beta power decrease are largely coextensive during action planning, they have been suggested to reflect different aspects of the action-triggering mechanism (Parés-Pujolràs et al., 2023). The RP has been modeled as the averaged output of a stochastic noisy accumulation process (Schurger et al., 2012), which triggers actions upon a threshold-crossing event. In turn, contralateral motor beta power is thought to reflect a threshold for action, which can be strategically adjusted (Steinemann et al., 2018; He et al., 2020) to either facilitate or hinder action initiation (Kelly et al., 2021).

However, investigations of the neural correlates of self-generated actions have been hampered by the difficulty of eliciting endogenous, self-generated actions in controlled laboratory settings (Haggard, 2008). As noted above, scientific work on self-generated actions has largely focused on “free choices” (e.g., “Move your wrist whenever you feel the urge to do so”; Libet et al., 1983) that are largely meaningless, in the sense of being unrelated to current goals. Some researchers have tried to enrich the notion of self-generated action, for example, by making self-generated actions trigger valenced outcomes (Maoz et al., 2019). Here we have taken a different approach to enriching self-generated action, based on embedding self-generated movements within a rich chain of actions that jointly represent a solution to a problem and thus a progression toward a meaningful goal. Participants made a series of actions that jointly constituted an endogenously generated solution to a problem set by the experimenter. Specifically, participants performed variants of the well-established Tower of London task (Shallice and Burgess, 1991). In one condition, they planned and then executed a sequence of actions to achieve a specified goal state (“self-generated”). In a control condition (“stimulus-driven”), they did not see any final goal but followed a set of imperative stimuli that led them one-by-one through the same set of moves.

Crucially, this manipulation isolates the contribution of internal action planning within a goal-directed context. In the self-generated condition, participants must construct a sequence of actions that achieves the goal state, engaging prospective planning processes. In contrast, the stimulus-driven condition provides step-by-step instructions that remove the need for internal sequence generation while preserving the sensory input, motor requirements, and overall task structure. The comparison between conditions therefore targets the neural mechanisms underlying self-generated action planning within problem-solving.

This contrast between self-generated, stimulus-independent and stimulus-driven actions has been widely studied (Jahanshahi et al., 1995; Praamstra et al., 1995; Dirnberger et al., 1998; Khalighinejad et al., 2019). Such studies typically report RPs only prior to self-generated actions. Some previous studies have investigated how different features of self-generated action influence the shape of the RP. Verleger et al. (2016) found that longer waiting times were associated with greater RP amplitudes and earlier onsets, suggesting that deliberation and preplanning enhance motor preparation signals. Travers et al. (2021) used a reinforcement-learning paradigm, allowing participants to learn the optimal time to make a self-generated action. As participants learned to plan the time of action RP amplitudes grew. Taken overall, previous results suggest that RP amplitude increases, and RP onset becomes earlier, in proportion to the presumed cognitive complexity of planning and executing the action.

To our knowledge, no previous EEG study has focused specifically on the links between self-generated actions and problem-solving. However, based on previous behavioral (Shallice, 1982) and RP studies (Travers et al., 2021), we predicted stronger (i.e., larger and/or earlier) RPs for problem-solving than for stimulus-driven actions and stronger RPs for the first action in a sequence, compared with later actions, reflecting the cognitive load of advanced planning. Similar predictions were made for beta-ERD.

Finally, to mitigate some of the concerns regarding the interpretation of RP signals (Schurger, 2018), we used some novel analysis steps. To avoid any unwarranted assumptions about when the cognitive processes of action generation begin, we adopted a no-baseline approach, using high-pass filtering at 0.05 Hz to remove slow drifts while preserving the temporal structure of preparatory activity. While the RP has been the major focus of this controversy, the same caveats presumably apply to any neural precursor signal, including, for example, beta-ERD. Furthermore, in addition to classical event-related potential analysis, we applied model-free classification, which avoids assumptions about the specific form of EEG components that may be relevant to the cognitive factors used in the experimental design.

## Materials and Methods

### Participants

Twenty-five healthy, right-handed participants (7 males, mean age 25.4 years, SD 4.9, range 19–35 years) participated in the study. Two participants were excluded from the EEG analysis due to excessive artifact-contaminated trials. None had a history of neurological or psychiatric disorders. Colorblind individuals were also excluded as the tasks crucially required detecting color differences. All inclusion and exclusion criteria were established before data collection commenced. All participants gave informed written consent. The study was approved by University College London Research Ethics Committee (approval code, 1825/003).

### Experimental task

We developed a modified, computerized version of the Tower of London task, a well-established paradigm frequently used to assess problem-solving abilities in healthy participants and neurological patients (Shallice and Burgess, 1991). The task presented participants with a series of two-dimensional arrangements of colored balls positioned on three pegs of different lengths. The aim was to transform the initial arrangement into a goal configuration by using the minimum number of moves, all while adhering to specific rules. Only one ball could be moved at a time. Each ball could be moved from one peg to another, with the constraint that no more than three, two, and one ball could be placed on the first, second, and third peg, respectively. If more than one ball was located in one peg, only the ball occupying the highest position could be selected (in line with the classical, physical version of the task).

In our modified task, the goal was presented on the right side of the screen and remained available until the goal achievement. The initial arrangement was presented on the left side of the screen and was progressively updated by participants’ actions on the tower configuration.

Each move within the tower configuration required the participant to perform two distinct button presses. The first button press selected the specific ball to be moved, determining the ball to be lifted from the peg. The second button press indicated the peg where the ball needed to be placed, determining the release of the selected ball on it.

Participants solved the problems using the index, middle, and ring fingers, with each finger corresponding to the selection of a specific peg, using the keyboard keys J, K, and L. Upon reaching the goal, participants were presented with a screen reporting the goal configuration on a gray background, indicating whether they successfully accomplished the problem within the minimal number of moves or not.

**Figure 1.**
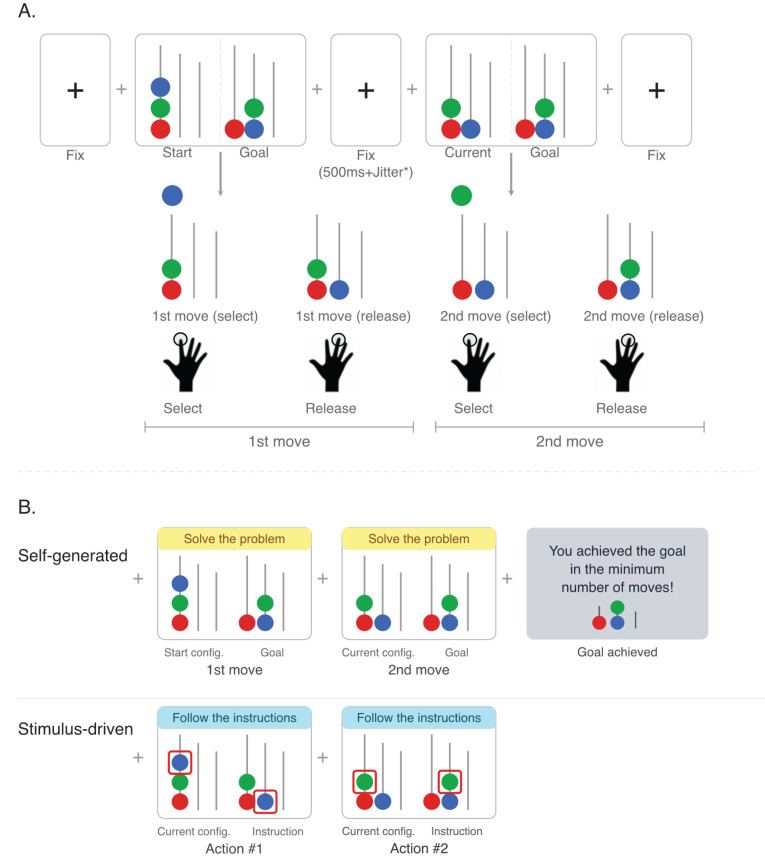
Investigating effects of cognitive context on self-generated action processes using the Tower of London task. ***A***, Schematic of the experimental task, based on a modified version of the Tower of London paradigm. Participants solved problems by moving colored balls across pegs with sequential keypresses, achieving a goal configuration with the minimum number of moves possible. ***B***, Experimental conditions. In the self-generated condition, participants could continuously see the goal configuration to achieve and chose which ball to move and where to place it at each step. In the stimulus-driven condition, no goal condition was shown. Instead, red rectangles appeared at each step to indicate which ball to select and where to place it next. An example of a simple problem requiring just two moves is shown.

A time interval of 250 ms plus a jitter interval ranging between 2.5 and 6.5 s was introduced between subsequent moves. During this time interval, a fixation cross was presented on the screen. The experiment was run using Psychtoolbox v.3.0 (Kleiner et al., 2007; Fig. 1*A*).

#### Problem selection and conditions

A total of 72 Tower of London problems were used, drawn from 12 distinct “families” expressed in six different color permutations. Half of the problems required two moves to be solved, while half required four optimal moves. The selection of problems was carried out using the Tower Tool software tool developed specifically for analyzing tower tasks (Kaller et al., 2011). Notably, all chosen problems had a singular optimal solution and were devoid of any detours or dead-ends.

The experimental consisted of six 10 min blocks. Within each block, problems were randomly selected while ensuring that problems from the same family were not included in the same run. Within each block, problems were randomly selected, with half of problems required “self-generated” solutions and half requiring participants to follow the instructed sequence of a “stimulus-driven” solution. Whether participants were in the self-generated or other-generated condition was indicated through a colored band positioned on the top of the screen—displaying the instruction “solve the problem” or “follow the instructions” (Fig. 1*B*).

Participants solved as many problems as possible in 10 min, earning an additional bonus of 0.5 pence for each problem completed in the minimum number of moves. This incentivized both accuracy and a reasonably short solution time for each problem.

### Procedure

The experiment started with obtaining participants’ informed consent, which was followed by a preliminary introduction and training phase before the actual recording. The training was structured into three phases. After reading the instructions, participants were asked to solve two problems, one requiring two moves and another involving four moves, under the intention-driven condition. Participants were then instructed to the stimulus-driven condition and asked to solve two additional problems, again consisting of two and four moves, but under the stimulus-driven condition. Subsequently, participants solved a mixed set of 12 problems including both intention-driven and stimulus-driven scenarios, presented in a randomized order.

After the experiment, participants were provided with a debriefing that explained the primary aims of the study and were compensation at a rate of £9.20 per hour.

### Behavioral data analysis

All analyses were performed by means of the statistical software R (4.0.3) and the lme4 package (Bates et al., 2014). RTs were log-transformed prior to analysis to reduce positive skewness. Fixed effects included sequence position (first move vs other moves), action type (self-generated vs stimulus-driven), and sequence length (two-move vs four-move), along with all two-way and three-way interactions. Random effects were specified by participant, with random intercepts and slopes for sequence position and action type (i.e., 1 + First + MovementType | ID). This random effects structure was selected as the most complex model that converged successfully from a series of models of increasing complexity. Fixed effects were tested using Type II Wald *χ*^2^ tests (car package). Post hoc comparisons were conducted using estimated marginal means (emmeans package). Specifically, targeted contrasts were used to test (1) whether the sequence position effect (first vs other moves) differed between action types across sequence lengths (difference-in-differences contrast) and (2) whether the first-move slowing differed between two-move and four-move sequences separately for each action type. All post hoc *p* values were corrected for multiple comparisons using the Bonferroni method.

The data and the R script for behavioral analysis are available at the following link: https://osf.io/c9yek/?view_only=4d853c7815024afc8db10f20579e7d73.

### EEG analyses

#### Preprocessing

EEG data were processed using MATLAB R2021a (MathWorks) and EEGLAB version 2021.0 (Delorme and Makeig, 2004).

The following steps were applied in the same order for both preprocessing variants described below. Data were rereferenced to the average of the two mastoid electrodes. A bandpass filter was applied to the continuous data (eighth-order Butterworth, zero-phase shift) with a 30 Hz low-pass cutoff; the high-pass cutoff differed between preprocessing variants as specified below. Data were downsampled to 200 Hz. Epochs of −1,000 to +500 ms relative to each key press were extracted. Independent component analysis (EEGLAB runica algorithm) was computed on the epoched data. Vertical and horizontal eye movement components were identified by visual inspection and removed. Finally, epochs with voltage fluctuations exceeding ±120 µV at any of the five frontocentral region-of-interest electrodes (Fz, FCz, Cz, FC1, FC2) were rejected.

The high-pass filter cutoff was set to 0.05 Hz to remove slow DC drifts while preserving the slow preparatory negativity of interest.

No baseline correction was applied to the EEG data. A key challenge in analyzing the RP is that the onset of preparatory activity is inherently difficult to define, particularly in paradigms with substantial variability in response timing (Schurger, 2018). Although RP analyses are typically baseline-corrected using a premovement interval, this procedure assumes that preparatory activity has not yet begun by that period. This assumption is questionable in contexts where preparation is highly desirable but is likely to be variable (Verbaarschot et al., 2015). Subtracting activity at or near movement onset (Khalighinejad et al., 2018) removes the very signal of interest and does so differentially across conditions in proportion to their RP amplitude at that timepoint. A fixation-period baseline is similarly problematic, as the jittered intertrial interval leads to variable baseline windows and may already contain condition-specific anticipatory activity. We therefore adopted a no-baseline approach. This approach avoids imposing assumptions about the absence of preparatory activity during a predefined baseline period. It also ensures consistency across ERP, β-band, and multivariate pattern analysis (MVPA) analyses and allows the full temporal evolution of preparatory signals to be characterized.

#### RP analysis

*Region of interest*. The RP was quantified as the mean voltage amplitude averaged across five frontocentral electrodes specified a priori: Fz, FCz, Cz, FC1, and FC2. This region of interest was chosen on the basis of the known scalp topography of the RP (Kornhuber and Deecke, 1965; Shibasaki and Hallett, 2006) and was not adjusted on the basis of the data.

*Factorial analysis of sequence position and sequence length*. The primary analysis compared self-generated and stimulus-driven waveforms in four conditions defined by the crossing of sequence length (two-move, four-move) and sequence position (first move, other moves). For the first move conditions, epochs were time-locked to the first key press of each trial. For the other moves conditions, epochs were time-locked to all subsequent key presses within a trial (Moves 2, 3, and 4 in four-move sequences; Move 2 only in two-move sequences).

To directly test the main effects and interaction of sequence position (first move vs other moves) and sequence length (two-move vs four-move), a 2 × 2 factorial cluster permutation analysis was applied to the self-generated minus stimulus-driven difference waveforms. Three effects were tested:

1. Main effect of sequence position: the advantage of first moves over other moves, averaged across both sequence lengths
2. Main effect of sequence length: the difference between four-move and two-move sequences, averaged across move positions
3. Sequence position by sequence length interaction: whether the first-move advantage is larger for four-move than for two-move sequences

Each contrast was submitted to a one-sample cluster permutation test (5,000 permutations; cluster-forming threshold *p* < 0.05; significance threshold *p* < 0.05, two-tailed).

*Direct comparison of the first-move signal between sequence lengths*. To directly test whether the self-generated preparatory advantage at the first move differed between two-move and four-move sequences, the self-generated minus stimulus-driven difference waveform at the first move of two-move sequences was compared against the equivalent waveform at the first move of four-move sequences (dependent-samples cluster permutation test, 1,000 permutations, cluster-forming threshold *p* < 0.05, significance threshold *p* < 0.05, two-tailed).

*Cluster analysis: four conditions*. In addition, for each of the four conditions, self-generated and stimulus-driven waveforms were compared using cluster-based permutation tests implemented using the FieldTrip toolbox (Oostenveld et al., 2011). At each time point in the analysis window (−1,000 to +500 ms relative to movement onset), a dependent-sample *t* statistic was computed across participants. Contiguous time points exceeding a cluster-forming threshold of *p* < 0.05 were aggregated into clusters by their summed *t* statistic (cluster mass), and each observed cluster mass was compared against the null distribution of maximum cluster masses from 1,000 Monte Carlo permutations of the condition labels. Clusters were considered significant at *p* < 0.05 (two-tailed).

*Move-position analysis: six conditions*. In a further analysis addressing the distinction between intermediate and final moves, trials from four-move sequences were separated by individual move position (first, second, third, and final move), yielding six position-specific condition pairs in total: the first move and the final move of two-move sequences and the first, second, third, and final moves of four-move sequences. Self-generated and stimulus-driven waveforms were compared separately at each of the six positions using the same custom cluster permutation procedure described above (1,000 permutations; cluster-forming threshold *p* < 0.05; significance threshold *p* < 0.05, two-tailed; *N* = 23).

*Linear trend across positions in four-move sequences*. To test for a monotonic decrease in the self-generated preparatory advantage across the four positions of four-move sequences, a linear trend contrast was applied. The self-generated minus stimulus-driven difference waveform was computed for each participant at each of the four positions. Linear contrast weights [1.5, 0.5, −0.5, −1.5] were applied across positions at each time point. The resulting contrast time series was submitted to a one-sample cluster permutation test (5,000 permutations; cluster-forming threshold *p* < 0.05; significance threshold *p* < 0.05, two-tailed; *N* = 23).

*Planned comparison: final versus nonfinal moves*. To directly test whether the self-generated preparatory negativity differed as a function of move position independently of sequence length, a planned comparison was conducted contrasting nonfinal moves against final moves, collapsing across sequence length. Nonfinal moves comprised the first move of two-move sequences and the first, second, and third moves of four-move sequences; final moves comprised the final move of two-move sequences and the final move of four-move sequences. For each participant, the self-minus-stimulus difference wave was averaged separately across nonfinal and final conditions, and the nonfinal minus final contrast was submitted to a one-sample cluster–based permutation test against zero (5,000 permutations; cluster-forming threshold *p* < 0.05, two-tailed).

*Reaction time and RP amplitude*. To assess whether differences in RP amplitude between self-generated and stimulus-driven actions could be accounted for by differences in reaction time (RT), we conducted two additional analyses. First, we ran a linear mixed model at the trial level with mean RP amplitude (averaged over the −1,000 to 0 ms premovement window across the significant cluster channels) as the outcome variable. RT (log-transformed, scaled within participant) was entered as a covariate alongside condition (self-generated vs stimulus-driven), move position (first vs other moves), and sequence length (two vs four moves), with all interactions included as fixed effects. The random effects structure included a random intercept and random slopes for condition and move position by participant. Model fit was assessed by the likelihood ratio test comparing models with and without RT. Pearson’s correlations between participant mean RT and participant mean RP amplitude were additionally computed separately within each condition (*N* = 23). Second, to directly equate RT distributions across conditions, we used a subsampling procedure in which self-generated trials were subsampled within each participant to match the RT distribution of stimulus-driven trials, using quantile binning (five bins). This procedure was repeated 1,000 times and results averaged across iterations. Mean RP amplitude was then compared between RT-matched self-generated trials and stimulus-driven trials using a paired *t* test across participants.

#### Beta-band power analysis

*Time–frequency decomposition*. Beta-band power was extracted using a separate preprocessing pipeline. The continuous EEG was epoched from −2,000 to +1,000 ms relative to each key press. No baseline correction was applied to the epoched data. Time–frequency representations were computed using a Hanning-tapered multitaper convolution (mtmconvol in FieldTrip) across 1–35 Hz in 1 Hz steps, with a fixed time window of 0.4 s applied at all frequencies. Power was averaged across the beta band (13–30 Hz) to yield a single time series per electrode per epoch and then averaged across trials within each condition per participant. Edge artifacts arising from the 0.4 s taper (∼40 samples at each end of the epoch) were set to NaN and excluded from all analyses.

*Region of interest*. The beta-band analysis used a region of interest comprising three left-hemisphere contralateral motor electrodes: Cz, C1, and C3, selected a priori on the basis of their sensitivity to motor preparation contralateral to the responding right hand. Power was averaged across these three electrodes before statistical analysis.

*Spectrogram visualization*. For display purposes, grand-average time–frequency spectrograms were computed across the full 1–35 Hz range using the same decomposition parameters. Raw (unnormalized) power was used, with a shared color axis computed from the 2^nd^ to 98th percentile of the data within the 13–30 Hz beta band across all conditions and the −1,000 to +500 ms window. Self-generated minus stimulus-driven difference spectrograms were computed by subtracting the grand-average stimulus-driven power from the grand-average self-generated power at each time–frequency point.

#### Statistical analysis

The statistical analysis followed the same structure as the RP analysis, with analyses conducted on the −998 to +499 ms window (the closest available approximation to −1,000 to +500 ms given the noninteger sampling resolution of the beta epoch). Three sets of analyses were conducted.

The 2 × 2 factorial analysis of sequence position (first move vs other moves) and sequence length (two-move vs four-move) was applied to the self-generated minus stimulus-driven difference in beta power, using the same custom cluster permutation procedure as the RP factorial (5,000 permutations; cluster-forming threshold *p* < 0.05, two-tailed; *N* = 22; one participant was excluded due to missing data in the two-move other moves condition).

A direct comparison of the self-generated minus stimulus-driven beta difference at the first move of two-move versus four-move sequences was conducted using a FieldTrip dependent-sample cluster permutation test (1,000 permutations; cluster-forming threshold *p* < 0.05, two-tailed; *N* = 23).

Pairwise comparisons of self-generated and stimulus-driven beta power for each of the four main conditions were conducted using FieldTrip dependent-sample cluster permutation tests (1,000 permutations; cluster-forming threshold *p* < 0.05, two-tailed; *N* = 23).

Move-position analyses were conducted using the same 12-condition data structure as the RP move-position analysis. Pairwise self- versus stimulus-driven comparisons were conducted at each of the six individual move positions using one-sample cluster permutation tests (1,000 permutations; cluster-forming threshold *p* < 0.05, two-tailed). A linear trend contrast across the four positions of four-move sequences was tested using orthogonal polynomial contrast weights [+1.5, +0.5, −0.5, −1.5] submitted to a one-sample cluster permutation test (5,000 permutations; cluster-forming threshold *p* < 0.05, two-tailed; *N* = 22). A planned comparison of nonfinal versus final moves, collapsing across sequence length, was conducted using the same one-sample cluster permutation test (5,000 permutations). Nonfinal moves comprised the first move of two-move sequences and the first, second, and third moves of four-move sequences; final moves comprised the final move of two-move sequences and the final move of four-move sequences.

#### MVPA

*Decoding procedure*. MVPA was used to test whether the spatial distribution of EEG activity could reliably distinguish self-generated from stimulus-driven actions on a trial-by-trial basis. Decoding was performed using linear discriminant analysis (LDA) implemented in the MVPA-Light toolbox (Treder, 2020). For each participant, the EEG data were taken from the no-baseline preprocessed files (high-pass 0.05 Hz, low-pass 30 Hz, downsampled to 200 Hz), from all EEG channels excluding EOG and occipital electrodes (O1, O2, POz) to minimize visual contributions, and from the −1,000 to 0 ms premovement window. At each timepoint, the spatial pattern across channels was used as the feature vector to classify the trial type (self-generated vs stimulus-driven), yielding a time series of decoding accuracy values. Accuracy was estimated using fivefold cross-validation repeated five times, with *z*-score normalization applied within each fold. The diagonal of the time × time generalization matrix was extracted, giving same-time decoding accuracy at each timepoint.

Decoding was performed separately for each of four conditions defined by sequence length and move position: two-move first move, two-move other moves, four-move first move, and four-move other moves.

*Statistical analysis*. Statistical significance of decoding accuracy against chance (0.5) was assessed using FieldTrip cluster-based permutation tests (one-sided, testing accuracy > chance; 5,000 permutations; cluster-forming threshold *p* < 0.05; significance threshold *p* < 0.025, equivalent to a two-tailed *α* = 0.05).

The factorial structure of the decoding results was tested using the same 2 × 2 custom cluster permutation procedure as the RP and beta analyses, applied to the decoding accuracy time series. The main effect of sequence position (first vs other moves), the main effect of sequence length (2-move vs 4-move), and their interaction were each tested using 5,000 permutations, cluster-forming threshold *p* < 0.05, and two-tailed significance threshold *p* < 0.05. Subjects with missing data in any condition were excluded from the factorial only.

*RT robustness check*. To assess whether MVPA decoding accuracy was driven by RT differences between conditions, all analyses were repeated after regressing out trial-level RT from the single-trial EEG patterns prior to classification. The RT was standardized within participant and regressed out of the EEG data at each channel and timepoint using ordinary least squares, retaining the trial mean (intercept) and removing only the RT-related variance (slope). The residualized EEG patterns were then passed to the same LDA classification pipeline as the primary analysis. The residualization was validated by confirming that the correlation between RT and EEG amplitude was reduced to zero after regression (*r* = 0.0000 for all participants).

The data and the R script for EEG analyses are available at the following link: https://osf.io/c9yek/?view_only=4d853c7815024afc8db10f20579e7d73.

## Results

### Manipulating self-generated actions in the Tower of London task

Our results showed that this manipulation produced clear behavioral signatures of action planning during self-generated trials.

To identify the optimal random effects structure for modeling RTs, a series of increasingly simplified linear mixed-effects models were tested, each including the fixed effects of Sequence position (First move vs Others), Condition (Self-generated vs Stimulus-driven), and Sequence length (2 vs 4), along with their interactions. Only one model, referred to as m2, successfully converged: RTs ∼ Sequence position × Condition × Sequence length + (1 + Sequence position + Condition | ID). This model included random intercepts and slopes for Sequence position and Condition on by participant (ID) and was retained for further analysis. More complex models specifying additional interaction terms or slopes for all fixed effects failed to converge, indicating overparameterization relative to the data structure.

The final model revealed a significant main effect of sequence position (*χ*^2^_(1)_ = 2,212.26; *p* < 0.0001), with substantially slower responses at the first move than at subsequent moves. There was also a significant main effect of sequence length (*χ*^2^_(1)_ = 127.83; *p* < 0.0001) and a significant main effect of action type (*χ*^2^_(1)_ = 36.43; *p* < 0.0001).

All two-way interactions were significant: sequence position × action type (*χ*^2^_(1)_ = 7,481.67; *p* < 0.0001), sequence position × sequence length (*χ*^2^_(1)_ = 86.86; *p* < 0.0001), and action type × sequence length (*χ*^2^_(1)_ = 189.18; *p* < 0.0001). A significant three-way interaction between sequence position, action type, and sequence length (*χ*^2^_(1)_ = 43.44; *p* < 0.0001) indicated that the difference in response times between first and subsequent moves varied depending on both the action type and sequence length.

**Figure 2.**
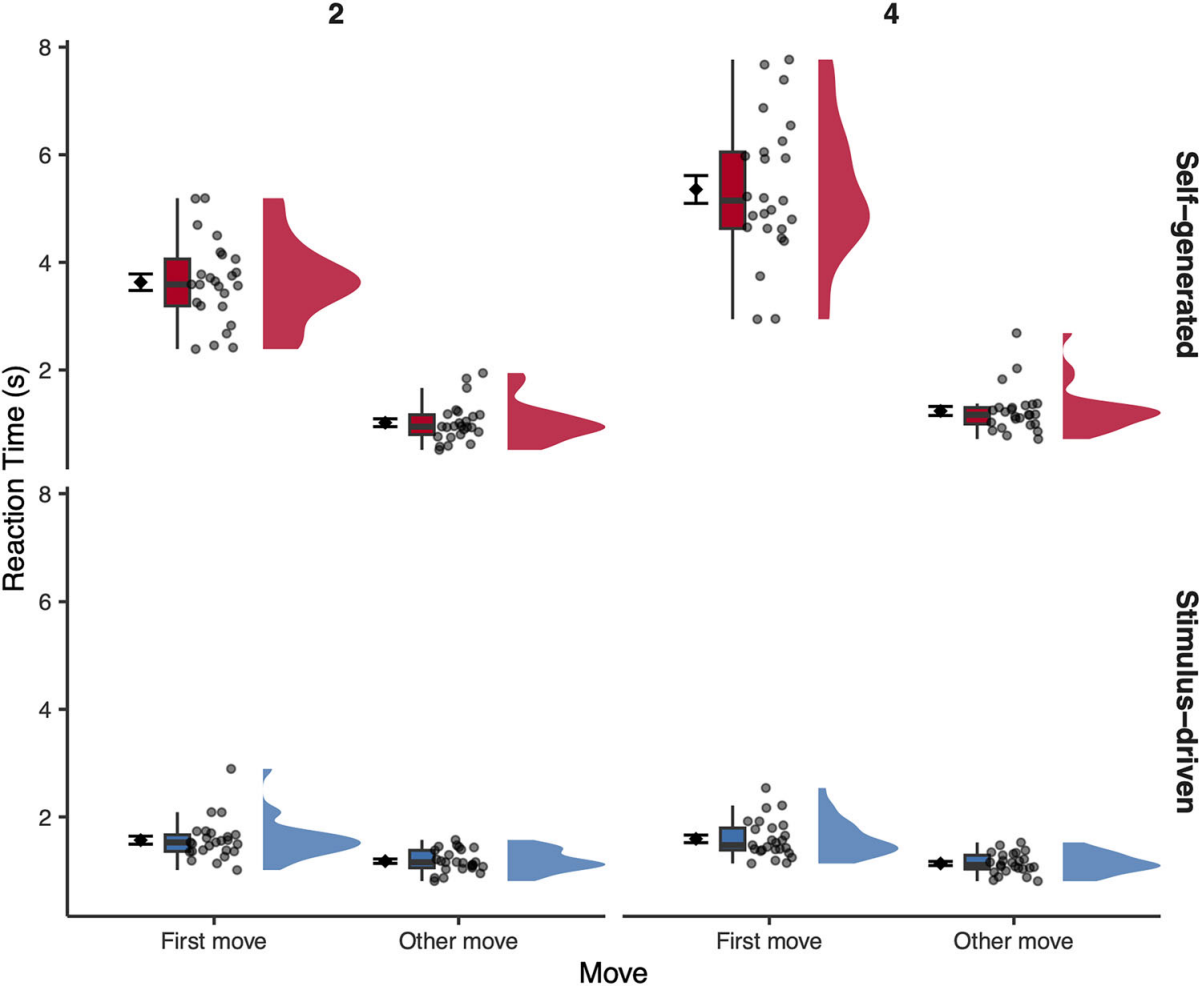
Response times for self-generated and stimulus-driven actions by move position and sequence length (*N* = 25). Each panel shows the distribution of mean response times (seconds) per participant for first moves and other moves, separately for two-move (left) and four-move (right) sequences and for self-generated (top, red) and stimulus-driven (bottom, blue) actions. Flat violins show the distribution of participant means; box plots show the median and interquartile range; individual points show participant means; the diamond symbol shows the grand mean ± SEM across participants. Raw RTs are shown for interpretability, but statistical analyses were performed on log-transformed RT values (Osborne, 2002).

A targeted contrast confirmed that the sequence position effect (first vs other moves) was significantly larger in the self-generated than in the stimulus-driven condition, and this difference increased with sequence length (estimate = −0.195; SE = 0.030; *z* = −6.59; *p* < 0.0001). Follow-up contrasts showed that in the self-generated condition, the first-move slowing was significantly larger for four-move than two-move sequences (estimate = 0.235; SE = 0.021; *z* = 11.26; *p* < 0.0001; two-move, first move *M* = 3.63 s; SD = 0.77; other moves *M* = 1.02 s; SD = 0.36; four-move, first move *M* = 5.36 s; SD = 1.29; other moves *M* = 1.24 s; SD = 0.42). In contrast, this increase was absent in the stimulus-driven condition (estimate = 0.040; SE = 0.021; *z* = 1.90; *p* = 0.057; two-move, first move *M* = 1.57 s; SD = 0.37; other moves *M* = 1.18 s; SD = 0.21; four-move, first move *M* = 1.59 s; SD = 0.35; other moves *M* = 1.13 s; SD = 0.19; Fig. 2).

### Neural correlates of self-generated action versus stimulus-driven action

We next examined how preparatory EEG signals differ between self-generated and externally cued actions, using three complementary neural measures: the RP, beta-band power, and MVPA.

We began by focusing on the RP. Electrodes Fz, FCz, Cz, FC1, and FC2 were selected a priori based on their established involvement in the RP (Shibasaki and Hallett, 2006; Travers et al., 2021).

Across analyses, the RP results converged on a clear and consistent pattern. Self-generated actions were associated with a more sustained preparatory negativity than stimulus-driven actions, and this effect was systematically modulated by sequence position.

#### Factorial analysis: sequence position and sequence length

*Main effect of sequence position*. A significant negative cluster spanning −1,000 to +495 ms (*p* = 0.0008; cluster mass = −1,154.72) indicated that first moves were associated with reliably greater self-generated preparatory negativity than other moves throughout the entire analysis window, averaged across both sequence lengths.

*Main effect of sequence length*. No significant clusters were found. The overall self-generated preparatory advantage did not differ between two-move and four-move sequences when averaged across move positions.

*Interaction*. No significant clusters were found (best *p* = 0.330). The first-move advantage was statistically equivalent for two-move and four-move sequences.

The factorial analysis thus reveals a single dominant structure in the RP data: a sustained preparatory advantage for first moves that is consistent in magnitude and time course across both sequence lengths, with no modulation by sequence length.

#### Direct comparison of the first-move signal between sequence lengths

A direct comparison of the self-generated minus stimulus-driven difference waveform at the first move of two-move versus four-move sequences revealed no significant clusters in either direction (no clusters found). The preparatory self-generated advantage at the first move was therefore statistically equivalent regardless of how many moves followed.

#### Cluster analysis: four conditions

Cluster-based permutation tests comparing self-generated and stimulus-driven waveforms at the frontocentral region of interest (Fz, FCz, Cz, FC1, FC2) revealed the following pattern across the −1,000 to +500 ms analysis window (*N* = 23).

*Two-move sequences, first move*. A significant negative cluster spanning −1,000 to +255 ms (*p* = 0.0004; cluster mass = −1,114.04) indicated greater preparatory negativity for self-generated relative to stimulus-driven actions throughout the premovement window and extending into the early postmovement period.

*Two-move sequences, other moves*. No significant clusters were found. The preparatory self-generated advantage was entirely absent at the other (final) move of two-move sequences.

*Four-move sequences, first move*. A significant negative cluster spanning −1,000 to +115 ms (*p* = 0.0002; cluster mass = −1,142.04) indicated a sustained preparatory self-generated advantage at the outset of four-move sequences, closely paralleling the two-move first move result.

*Four-move sequences, other moves*. No significant clusters were found (best *p* = 0.1714). When Moves 2, 3, and 4 are averaged together as a single “other moves” condition, the self-generated preparatory advantage does not reach significance. As shown below, this null result reflects the averaging of a significant effect at Move 2 with null or reversed effects at Moves 3 and 4, motivating the move-position analysis.

**Figure 3.**
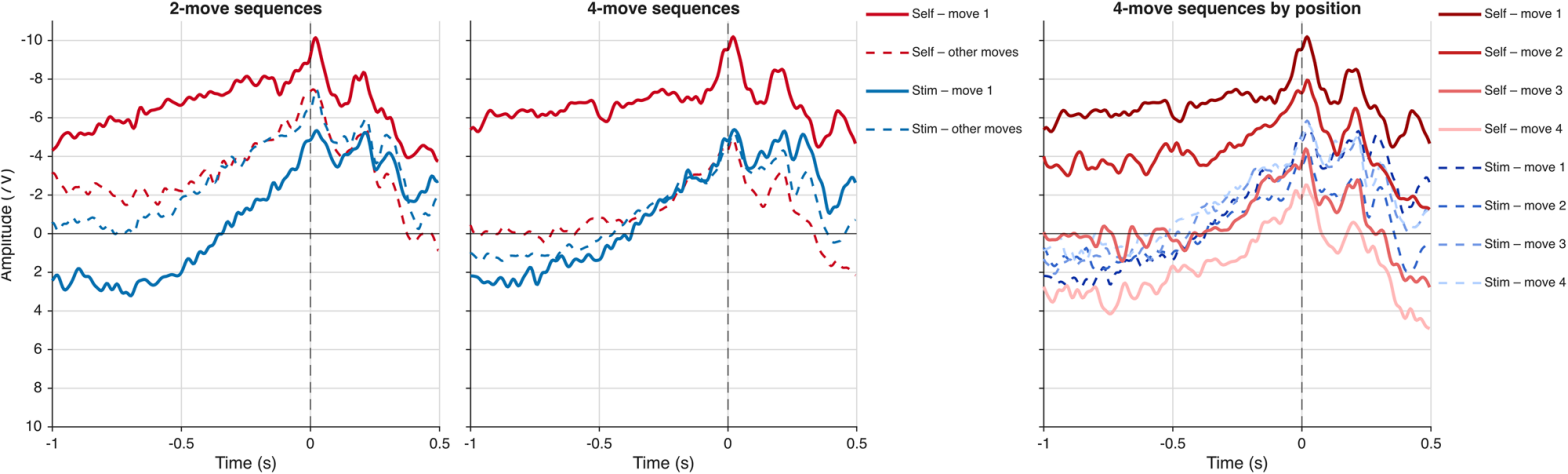
RP waveforms: no-baseline analysis (*N* = 23). Grand-average RP amplitudes at the frontocentral region of interest (Fz, FCz, Cz, FC1, FC2) for self-generated (red) and stimulus-driven (blue) actions. Left panel, two-move sequences; mid panel, four-move sequences. Solid lines indicate first moves; dashed lines indicate other moves (final move of two-move sequences; Moves 2–4 averaged for four-move sequences). Left panel, four-move sequences. Self-generated waveforms are shown in a red gradient (dark to light, Moves 1–4); stimulus-driven waveforms are shown in a blue gradient (dark to light, Moves 1–4). Solid lines, self-generated; dashed lines, stimulus-driven. A monotonic decrease in the self-generated preparatory negativity from Move 1 to Move 4 is visible in the right panel and was confirmed by a significant linear trend across positions (cluster-based permutation test, *p* < 0.0001). Negative amplitude is plotted upward. Time 0 indicates movement onset (dashed vertical line). Analysis window: −1,000 to +500 ms relative to movement onset.

The four-condition analysis therefore reveals a clear and consistent first-move advantage: the RP was significantly enhanced for self-generated relative to stimulus-driven actions at the first move of both sequence lengths (Fig. 3).

#### Move-position analysis: six conditions

To further unpack the role of sequence position, we conducted a more fine-grained move–position analysis that treated each move within the sequence separately (two-move: first, second; four-move: first, second, third, fourth). This analysis revealed a striking and systematic pattern. The self-generated preparatory advantage was present at all nonfinal moves but was markedly reduced and in some cases reversed at the final move, regardless of sequence length.

Separating the other moves of four-move sequences into individual positions revealed a progressive decrease in the self-generated preparatory advantage from the first to the final move.

#### Cluster analysis: six conditions

*First move of two-move sequences*. A significant negative cluster spanned −1,000 to +215 ms (*p* < 0.001), indicating a large and sustained preparatory self-generated advantage throughout the premovement window.

*Final move of two-move sequences*. No significant clusters were found. The preparatory advantage was entirely absent at the final (second) move.

*First move of four-move sequences*. A significant negative cluster spanned −1,000 to +185 ms (*p* < 0.001), closely mirroring the first move of two-move sequences.

*Second move of four-move sequences*. Significant negative clusters spanned approximately −950 to +70 ms (*p* < 0.05), indicating a preparatory advantage that was reduced in extent compared with the first move.

*Third move of four-move sequences*. No significant negative clusters were found. The self-generated waveform was more negative throughout the preparatory window in the expected direction, but no time points reached the cluster-forming threshold for a negative cluster. Only nonsignificant positive single-timepoint clusters were identified.

*Final move of four-move sequences*. No significant preparatory negative cluster was found. Instead, significant positive clusters were present in the postmovement window (+85 to +495 ms; *p* < 0.05), indicating that stimulus-driven responses were associated with greater postmovement negativity than self-generated responses at the final move. Scattered significant positive clusters were also present at some premovement timepoints but are not considered interpretable given their isolated single-timepoint character and the absence of a coherent preparatory pattern.

Together, the move-position analysis reveals that the key distinction is between final and nonfinal moves, irrespective of sequence length. The self-generated preparatory advantage is present at all nonfinal moves (first, second and third of four-move sequences; first of two-move sequences) and absent or reversed at the final move of both sequence lengths. This is precisely what would be expected if the preparatory neural signal reflects the degree of endogenous forward planning required: planning demands decrease as the sequence approaches its only remaining move, where matching the current board state to the target configuration is sufficient to guide the action.

#### Linear trend across positions in four-move sequences

This progressive change across the sequence was formally tested using a linear trend analysis in the four-move condition. The results confirmed a highly significant monotonic decrease in the self-generated preparatory advantage from the first to the final move (*p* < 0.0001; cluster mass = −1,779.65; −1,000 to +495 ms), indicating that preparatory activity became progressively less self-specific as the sequence unfolded (Fig. 3, right panel).

#### Planned comparison: final versus nonfinal moves

To directly test the distinction between early and late sequence stages, we conducted a planned comparison collapsing across sequence length. This contrast revealed that nonfinal moves were associated with significantly greater self-generated preparatory negativity than final moves across the entire analysis window (*p* < 0.0001; cluster mass = −1,705.28; −1,000 to +495 ms).

#### RT and RP amplitude

To rule out the possibility that the larger RP for self-generated relative to stimulus-driven actions was driven by the corresponding difference in RTs, we conducted two analyses. In a mixed model including RT as a covariate, RT significantly improved model fit (*χ*^2^_(1) _= 36.5; *p* < 0.001), confirming that RT and RP amplitude covary. However, condition remained a highly significant predictor of RP amplitude after controlling for RT (*χ*^2^_(1)_ = 48; *p* < 0.001), with all other fixed effects remaining significant as in the main analysis. Within-condition correlations between participant mean RT and mean RP amplitude were nonsignificant for both self-generated actions (*r* = 0.07; *p* = 0.766) and stimulus-driven actions (*r* = 0.33; *p* = 0.119), indicating that RT does not predict RP amplitude within either condition. In the RT-matched subsampling analysis, mean RTs were virtually identical across conditions following subsampling (self-generated, *M* = −0.023; stimulus-driven, *M* = −0.030), confirming successful matching. Nevertheless, RP amplitude remained significantly more negative for self-generated than stimulus-driven actions (*t*_(22)_ = −3.51; *p* = 0.002; *d* = −0.73). Taken together, these results demonstrate that the RP difference between conditions reflects a genuine difference in neural motor preparation that is not reducible to differences in RT.

We next examined contralateral motor β-band power (13–30 Hz) in response-locked epochs (−1 to 0.5 s) at electrodes Cz, C1, and C3, given that all responses were performed with the right hand. As in the RP analysis, we analyzed each combination of sequence length (2 vs 4 moves) and move type (first vs other) separately.

The resulting pattern closely paralleled the RP findings. Self-generated actions were associated with an early and pronounced β desynchronization, whereas stimulus-driven actions and later sequence steps showed reduced suppression or transient postmovement rebounds. Critically, the magnitude of this self-versus-stimulus difference depended on sequence structure.

#### Factorial analysis: sequence position and sequence length

*Main effect of sequence position*. A significant negative cluster spanning −392 to −157 ms (*p* = 0.039; cluster mass = −119.72; *N* = 22) indicated greater beta suppression for first moves than for other moves in the premovement window, averaged across both sequence lengths. This effect is consistent with the RP position effect, though it emerged later in the preparatory window and was more circumscribed in time.

*Main effect of sequence length*. No significant clusters were found (best *p* = 0.269). The overall self-generated beta suppression advantage did not differ between two-move and four-move sequences when averaged across move positions.

*Interaction*. A significant positive cluster was found in the postmovement window, spanning +68 to +229 ms (*p* = 0.030; cluster mass = +97.88; *N* = 22). This interaction reflects a crossover in the postmovement period: for two-move sequences, other moves (i.e., the final move) showed greater self-generated beta suppression than first moves postmovement (mean difference = −0.185 µV^2^), whereas for four-move sequences, first moves showed greater suppression than other moves (mean difference = +0.200 µV^2^). This pattern reflects postmovement beta dynamics rather than preparatory differences. At the final move of two-move sequences, the sequence is complete, and self-generated actions show an early beta rebound relative to stimulus-driven actions, reducing the self-generated suppression advantage. At nonfinal moves of four-move sequences, the sequence continues postmovement, and beta suppression for self-generated actions remains sustained. The interaction therefore captures the presence or absence of continued planning demands following each move rather than a genuine modulation of the preparatory position effect by sequence length.

#### Direct comparison of the first-move signal between sequence lengths

A direct comparison of the self-generated minus stimulus-driven beta suppression difference at the first move of two-move versus four-move sequences revealed no significant clusters in either direction (best *p* = 0.288). The preparatory self-generated beta suppression advantage at the first move was therefore statistically equivalent regardless of sequence length, consistent with the RP result.

#### Cluster analysis: four conditions

Cluster-based permutation tests comparing self-generated and stimulus-driven beta power at the contralateral motor region of interest (Cz, C1, C3) revealed the following pattern across the −998 to +499 ms analysis window (*N* = 23).

*Two-move sequences, first move*. Significant negative clusters spanned −998 to −973 ms (*p* = 0.002–0.044, Self < Stim), indicating a brief but reliable early premovement self-generated beta suppression advantage at the very start of the analysis window. The effect was limited to ∼25 ms at the earliest part of the window, suggesting that the sustained suppression visible in the waveforms across the full premovement period did not reach significance as a coherent cluster, likely due to the fragmented single-timepoint nature of the FieldTrip clustering applied here.

*Two-move sequences, other moves*. No significant clusters were found (best *p* = 0.052). The self-generated beta suppression advantage was absent at the other (final) move of two-move sequences within the analysis window.

*Four-move sequences, first move*. Significant negative clusters spanned −963 to −337 ms (*p* = 0.002–0.040, Self < Stim), indicating a sustained and broad premovement self-generated beta suppression advantage at the outset of four-move sequences.

**Figure 4.**
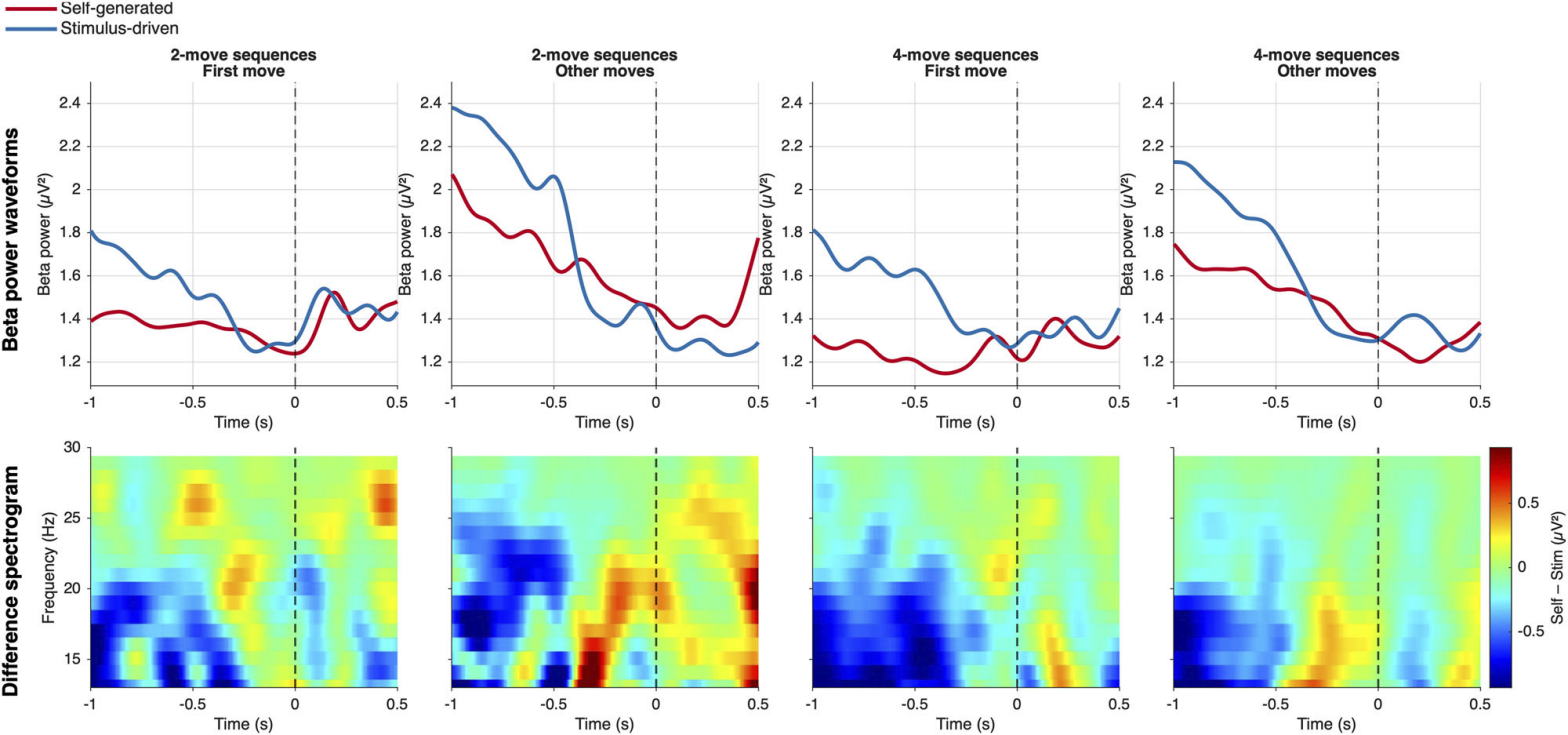
Beta-band power (13–30 Hz) at contralateral motor electrodes (Cz, C1, C3) for self-generated and stimulus-driven actions (*N* = 23). Top row, Grand-average beta power time series for self-generated (red) and stimulus-driven (blue) actions, time-locked to movement onset (dashed vertical line), across four conditions defined by sequence length (two-move, four-move) and move position (first move, other moves). Bottom row, Self-generated minus stimulus-driven difference spectrograms across the beta band (13–30 Hz), with the jet color scale indicating greater beta suppression for self-generated (blue) relative to stimulus-driven (red/yellow) actions. Beta power was extracted using a Hanning-tapered multitaper convolution across 1–35 Hz with a fixed 0.4 s time window; no baseline correction was applied. Analysis window: −1,000 to +500 ms relative to movement onset.

*Four-move sequences, other moves*. Significant negative clusters spanned −998 to −503 ms (*p* = 0.008–0.050, Self < Stim), indicating a sustained premovement self-generated beta suppression advantage when Moves 2, 3, and 4 of four-move sequences are averaged together. This contrasts with the two-move other moves null result, suggesting that beta suppression for self-generated actions persists across nonfinal positions within four-move sequences even when those positions are not the first move (Fig. 4).

The temporal profile of β power differed qualitatively from that of the RP. Notably, β power was already strongly suppressed up to 1 s prior to the first move in self-generated sequences and did not exhibit a gradual ramp-like decrease as movement approached. This suggests that, unlike the RP, β desynchronization may not reflect an accumulative preparatory process. Instead, it may index a strategic state set well in advance of action execution. In this framework, a motor plan may be established early and maintained via sustained β suppression, while the RP reflects a separate triggering process that brings the plan to execution. We return to this distinction in Discussion.

A key difference from the RP analysis emerged when examining β suppression across individual move positions. Whereas the RP effect was restricted to nonfinal moves, β suppression was present at all six move positions, including the final moves of both sequence lengths. This suggests that β desynchronization reflects a broader motor engagement signal that persists even when forward planning is no longer required.

This was confirmed by a move-position analysis that treated each step in the sequence separately.

#### Move-position analysis: six conditions

Pairwise cluster permutation tests comparing self-generated and stimulus-driven beta power at each of the six move positions revealed significant negative clusters (Self < Stim, indicating greater beta suppression for self-generated actions) at all six positions, though differing in temporal extent and magnitude.

*First move of two-move sequences*. A significant negative cluster spanned −998 to −553 ms (*p* < 0.0001; mass = −247.05), indicating sustained premovement self-generated beta suppression across much of the preparatory window.

*Final move of two-move sequences*. A significant negative cluster spanned −928 to −653 ms (*p* = 0.023; mass = −159.30), indicating early premovement self-generated beta suppression even at the final move of two-move sequences. This contrasts with the RP, where no significant preparatory advantage was found at this position, and suggests that beta suppression persists at the final move, albeit in a narrower and earlier window than at the first move.

*First move of four-move sequences*. A significant negative cluster spanned −998 to −277 ms (*p* < 0.0001; mass = −571.43), indicating the strongest and most sustained preparatory self-generated beta suppression of all six positions, consistent with the greatest planning demands at the outset of the longest sequences.

*Second move of four-move sequences*. Two significant negative clusters were found: a premovement cluster spanning −998 to −678 ms (*p* = 0.014; mass = −224.45) and a postmovement cluster spanning +53 to +279 ms (*p* = 0.041; mass = −152.92). The premovement cluster indicates sustained early preparatory suppression, and the postmovement cluster likely reflects continued motor planning for the remaining moves in the sequence.

*Third move of four-move sequences*. A significant negative cluster spanned −998 to −533 ms (*p* = 0.002; mass = −249.79), indicating a sustained premovement self-generated beta suppression advantage at this intermediate position.

*Final move of four-move sequences*. A significant negative cluster spanned −998 to −783 ms (*p* = 0.023; mass = −134.65), indicating early premovement self-generated beta suppression even at the last move of four-move sequences, though restricted to the earliest part of the preparatory window.

Unlike the RP, which showed a clear absence of preparatory advantage at final moves, beta suppression remained present across all six positions. This suggests that while the RP specifically indexes the slow buildup of preparatory neural activity that is absent when no further planning is required, beta suppression reflects a broader motor engagement signal that persists even at sequence-ending moves.

#### Linear trend across positions in four-move sequences

Although the magnitude of the self-generated advantage showed a numerically monotonic decrease across positions within four-move sequences (C1 = −0.297; C2 = −0.227; C3 = −0.181; C4 = −0.128 µV^2^), a formal linear trend test did not reach significance (best *p* = 0.299). This likely reflects the reduced sensitivity of β power compared with the RP, which exhibited a clear graded decline across the sequence.

#### Planned comparison: final versus nonfinal moves

**Figure 5.**
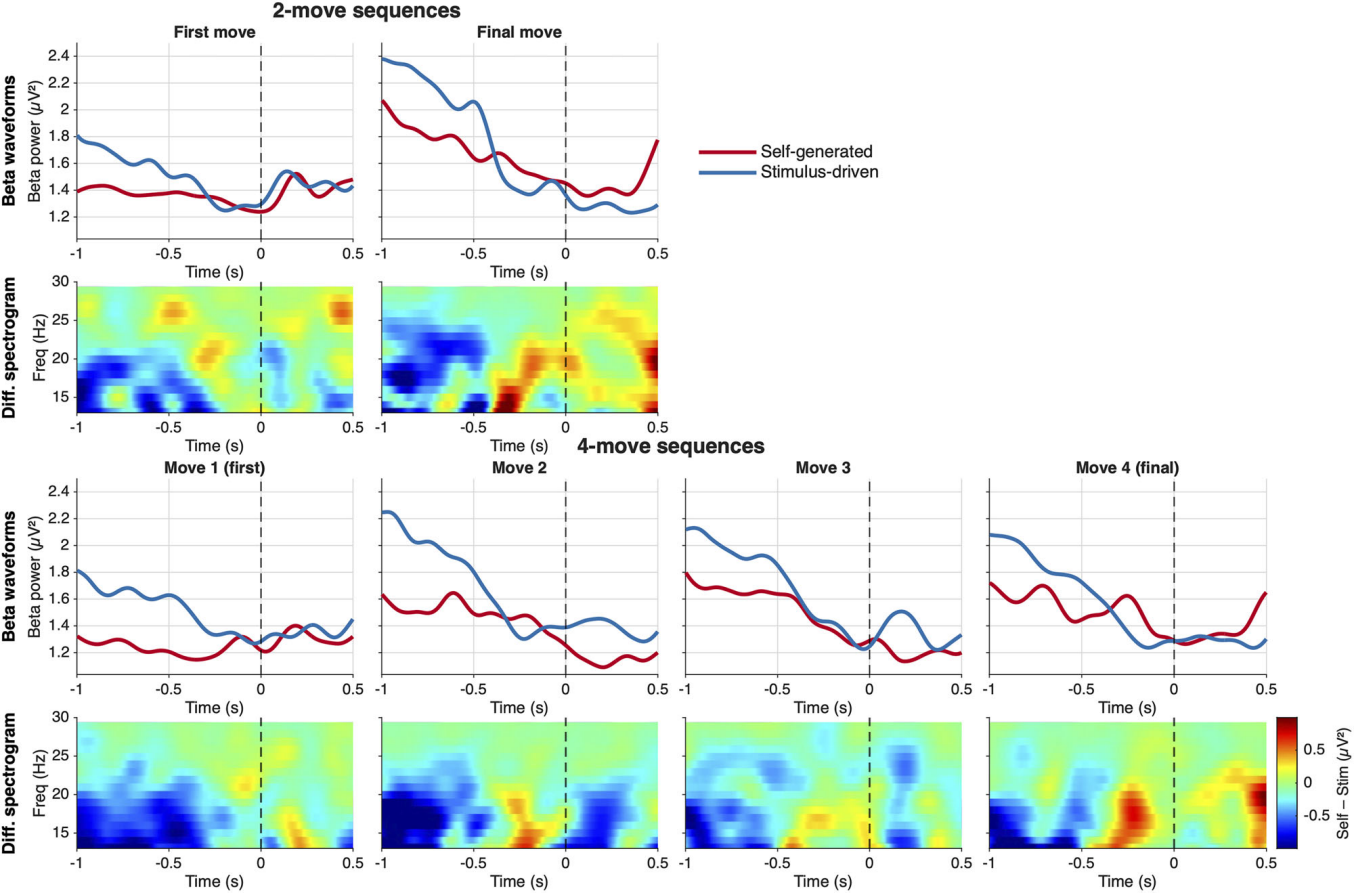
Beta-band power (13–30 Hz) at contralateral motor electrodes (Cz, C1, C3) by move position for self-generated and stimulus-driven actions (*N* = 23). Top block (two-move sequences), grand-average beta power time series (top panels) and self-generated minus stimulus-driven difference spectrograms across the beta band (top and bottom panels, respectively) for the first and final moves of two-move sequences. Bottom block (four-move sequences), equivalent plots for each of the four individual move positions. In the waveform panels, red, self-generated; blue, stimulus-driven; the dashed vertical line marks movement onset. In the difference spectrograms, the jet color scale indicates greater beta suppression for self-generated relative to stimulus-driven actions (blue) or the reverse (red/yellow); color axis is shared across all six difference maps (microvolts squared). Beta power was extracted using a Hanning-tapered multitaper convolution (1–35 Hz, 0.4 s fixed window) with no baseline correction applied.

The planned comparison of nonfinal versus final moves did not yield a significant premovement cluster. A significant negative cluster was found in the postmovement window, spanning +23 to +314 ms (*p* = 0.009; mass = −153.09), indicating that nonfinal moves were associated with greater self-generated beta suppression than final moves after movement onset. This postmovement effect mirrors the interaction observed in the factorial analysis and reflects the continued motor planning demands that follow nonfinal moves: after executing a nonfinal move, beta suppression remains elevated as the next move is planned, whereas after a final move, beta begins to recover. The absence of a significant premovement difference between final and nonfinal moves stands in contrast to the RP result, reinforcing the interpretation that these two signals capture distinct aspects of motor preparation. Taken together, these findings reinforce a functional dissociation between the two signals. Whereas the RP appears to track the progressive reduction in forward planning demands across a sequence, β suppression indexes a more sustained state of motor engagement that is maintained throughout action execution, even when planning requirements have been resolved (Fig. 5).

We complemented our univariate analyses with time-resolved, scalp-wide decoding of self-generated versus stimulus-driven actions, excluding occipital electrodes (O1, O2, POz) to minimize visual contributions. If preparatory activity carries condition-specific information, multichannel EEG patterns should discriminate between conditions prior to movement.

**Figure 6.**
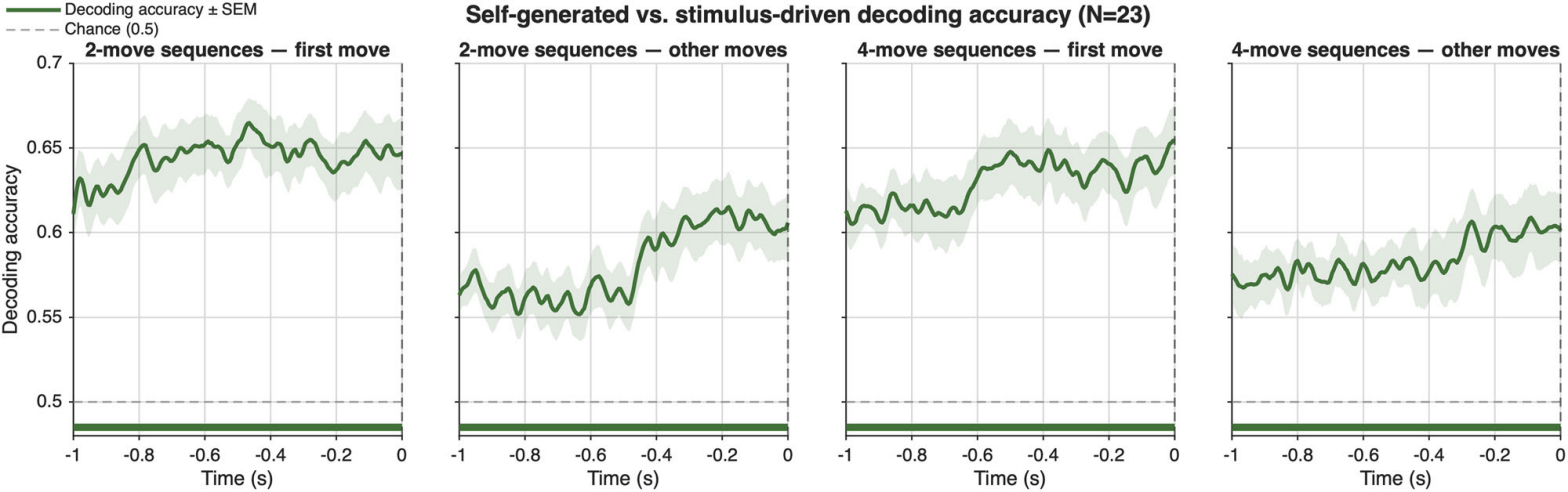
Multivariate decoding of self-generated versus stimulus-driven actions across the premovement window. Each panel shows the temporal profile of LDA decoding accuracy for distinguishing self-generated from stimulus-driven actions, separately for four conditions defined by sequence length (two-move, four-move) and move position (first move, other moves). Shaded regions indicate ±1 SEM across participants. The dashed horizontal line marks chance-level performance (0.5). The solid horizontal bar at the bottom of each panel indicates the time window of significant above-chance decoding (cluster-based permutation test, one-sided, *p* < 0.025; 5,000 permutations). Decoding was performed using the spatial pattern across 23 EEG channels in the −1,000 to 0 ms premovement window, with fivefold cross-validation repeated five times.

To test this, we used MVPA-Light to train four independent binary LDA classifiers (“two-move first move,” “two-move other moves,” “four-move first move,” “four-move other moves”) on data from −1 to 0 s relative to keypress. Classification performance was estimated using fivefold cross-validation with five repeats, yielding robust trial-wise accuracy estimates. Each classifier was trained to distinguish self-generated from stimulus-driven actions within the corresponding condition (Fig. 6).

#### Factorial analysis: sequence position and sequence length

*Main effect of sequence position*. A significant positive cluster spanning −1,000 to −325 ms (*p* = 0.0021; cluster mass = +419.36) indicated that decoding accuracy was reliably higher for first moves than for other moves across a broad premovement window, averaged across both sequence lengths. This result mirrors the RP position effect and indicates that the spatial distribution of EEG activity more reliably distinguishes self-generated from stimulus-driven actions at the first move of a sequence than at subsequent moves.

*Main effect of sequence length*. No significant clusters were found. Decoding accuracy did not differ overall between two-move and four-move sequences when averaged across move positions, consistent with the RP and beta factorial results.

*Interaction*. No significant clusters were found (best *p* = 0.4584).

#### Cluster analysis: four conditions

Cluster-based permutation tests comparing decoding accuracy against chance (0.5) revealed significant above-chance decoding throughout the entire premovement window in all four conditions (all *p* = 0.0002), spanning −1,000 to 0 ms. Self-generated and stimulus-driven actions were therefore reliably distinguishable from each other based on the spatial distribution of EEG activity across the full second preceding movement onset, regardless of sequence length or move position. This finding indicates that the preparatory neural signal distinguishing self-generated from stimulus-driven actions is present and decodable across all conditions and is not limited to specific time windows or move positions within the −1,000 to 0 ms epoch.

The uniformly significant decoding across all four conditions, combined with the significant position main effect in the factorial analysis, indicates that while preparatory information is present throughout the premovement window in all conditions, the strength of that information—as indexed by decoding accuracy—is modulated by move position: first moves carry more discriminative information than other moves.

#### RT robustness check

To assess whether MVPA decoding accuracy was driven by RT differences between conditions, all analyses were repeated after regressing out trial-level RT from the single-trial EEG patterns prior to classification (see Materials and Methods). After RT residualization, pairwise decoding against chance remained significant across the full premovement window for all four conditions (all *p* = 0.0002), confirming that above-chance decoding does not depend on RT differences between self-generated and stimulus-driven trials. In the factorial analysis, the main effect of position did not survive RT residualization (best *p* = 0.503). Instead, a significant interaction between position and sequence length emerged (two negative clusters, −845 to −575 ms; *p* = 0.003; mass = −186.18; −565 to −430 ms; *p* = 0.018; mass = −86.05), indicating that after RT removal, the position effect differs across sequence lengths: for two-move sequences, first moves remained more decodable than other moves (mean accuracy difference = +0.019), whereas for four-move sequences, this pattern reversed (mean accuracy difference = −0.037). A significant main effect of length was also observed (three negative clusters spanning −430 to −80 ms; all *p* < 0.05), indicating greater decodability for two-move than two-move sequences after RT removal. These results indicate that the primary decoding result (reliable above-chance classification in all four conditions) is fully robust to RT differences, while the factorial position effect reflects a combination of genuine neural differences in motor preparation and differences in planning time between first and other moves.

Taken together, these decoding results extend the univariate findings by showing that condition-specific preparatory information is not only present but also distributed across the scalp and reliably decodable at the single-trial level. Importantly, the modulation of decoding accuracy by sequence position mirrors the effects observed in both the RP and β analyses, with stronger discrimination at the start of the sequence and reduced differentiation at later stages; however, after RT residualization this position gradient was significant only for two-move sequences. This convergence suggests that the preparatory advantage for self-generated actions reflects a robust and spatially distributed neural signature, which is most pronounced when actions must be internally generated and becomes less distinct as the sequence unfolds and behavior becomes increasingly constrained by external structure.

## Discussion

The capacity to decide and generate one's own course of action is central to the ability to solve complex problems in the real world. Interestingly, this distinctive feature of human mental life has tended to fall between two stools in psychology and neuroscience research. While executive function studies have long investigated cognitive processes of problem-solving (Norman and Shallice, 1986; Goel and Grafman, 1995; Cooper and Shallice, 2000; Daw et al., 2005; Wagner et al., 2006; Mushiake et al., 2009; Cisek and Kalaska, 2010; Gonen-Yaacovi et al., 2013), this literature has generally neglected the role of self-generated actions in executing those solutions. Problem-solving is considered a purely cognitive process of reasoning, with the motor implementation of the solution being delegated to lower-level execution modules. Conversely, the cognitive neuroscience of self-generated actions has largely ignored the fact that self-generated actions typically occur as individual elements in a chain of purposive behavior that solves a current problem. That is, humans and other animals make self-generated actions because they have reasons for acting and goals that they can achieve through their actions (Haggard, 2019). In contrast, self-generated actions research has largely focused on “free” choices and initiation of simple movements in the absence of external pressures—perhaps reflecting an inheritance from the metaphysical concept of “free will.” Recent focus on enriching the neuroscience of self-generated action has focused on value rather than complexity (Maoz et al., 2019), while recent research on complexity of human action sequences has generally favored instructed rather than self-generated actions (Koechlin and Hyafil, 2007).

Our study represents a first step to fill the gap between these two traditions. We begin with the strong assumption that self-generated actions often represent solutions to problems. We then show that the classic neural precursors of self-generated action are enhanced in cases when an action is the output of a problem-solving process that this enhancement is focused on the preplanning stage rather than the execution stage of the problem sequence. That is, these effects were strongest prior to the first move in a problem sequence, compared with other moves.

In our version of the Tower of London task, participants generated a series of self-guided action steps that progress toward a final goal. The actions were (pairs of) keypress actions, of the kind used previously in self-generated actions experiments. At each step, participants autonomously selected from multiple possible actions, and advanced toward a goal that was specified for each problem. Importantly, the representation of the goal was not always sufficient to specify which action to make next. That is, there were always multiple ways to achieve the goal configuration of the Tower of London, though some solutions were of course more efficient than others. Therefore, participants’ actions were clearly up to them or internally generated.

We also developed a “stimulus-driven” control condition in which an imperative stimulus told participants at each step which ball to move to which peg, without any representation of an overall goal nor requirement to choose or plan any move. Such contrasts between self-generated and stimulus-driven actions have classically been used to identify the processes underlying self-generated action (Passingham, 1987; Jahanshahi et al., 1995; Jenkins et al., 2000; Seghezzi et al., 2019). Our design allowed us to estimate this same classical contrast in the context of complex, “intelligent” problem-solving behaviors having a clear link to cognitive theories of intelligence (Shallice, 1982). This design provides a unique window into goal-directed, meaningful actions that remain largely self-generated and minimally influenced by external stimuli, preserving the intrinsic nature of self-generated decision-making within a structured, goal-oriented framework.

Critically, RPs for self-generated actions were characterized by an earlier and more sustained buildup of preparatory activity compared with the later-onset ramps observed in stimulus-driven actions. These differences were most pronounced at the initial move of a sequence. Consistent with the factorial and cluster-based analyses, the self-generated preparatory advantage was restricted to the first move and was absent at subsequent moves in both two- and four-move sequences. In particular, RPs for later moves were comparable across conditions, suggesting that once a sequence is underway, action selection can be guided more directly by the current state of the task. This pattern suggests that the neural processes distinguishing self-generated from stimulus-driven actions are most strongly engaged at the point where an action sequence must be internally generated rather than during its subsequent execution. More generally, internal and stimulus-guided systems for action appear to diverge most strongly when actions must be generated in advance of execution rather than being directly specified by current inputs.

A potential concern is whether the larger RP for self-generated relative to stimulus-driven actions simply reflects longer RTs, rather than qualitatively different neural preparation. We addressed this in two ways. Conceptually, RT and RP are not independent variables where one confounds the other but covarying expressions of the same underlying planning process: volitional action selection both takes longer and recruits greater preparatory neural activity than stimulus-driven movement. This interpretation is consistent with the established role of the RP as a marker of motor preparation duration and intensity (Shibasaki and Hallett, 2006). Statistically, condition remained a significant predictor of RP amplitude after controlling for RT in a mixed model, and the RP difference persisted after RT-matched subsampling, indicating that the effect cannot be explained solely by differences in RT.

Several previous studies have investigated how the RP is related to cognitive and motoric features of action. On the one hand, the RP was found to increase with motor vigor (e.g., muscle contraction force; Kristeva et al., 1990). On the other hand, increasing attention to action also increases RP amplitudes. Thus, Libet and colleagues noted that RP amplitudes were systematically greater when participants reported carefully preplanning when to move, compared with when they did not (Libet et al., 1983). RPs typically have greater amplitude before choice actions than before simple actions (Jahanshahi et al., 1995). Our result broadly confirms this importance of the cognitive processes leading up to action and provides a unifying explanation for them. The RP amplitude appears sensitive to the cognitive processes that allow simple movements to organize into more goal-directed action sequences that achieve a desired state (Koechlin and Summerfield, 2007). Our findings therefore contrast with recent work that analyzes the RP in the context of accumulator models. Those studies typically use a theoretical framework of discrete decisions based on perception-like evidence (O'Connell and Kelly, 2021) and do not aim to focus on complex behaviors. Previous work on self-generated action precursors generally aimed to identify an initial decision to act. Our approach, in contrast, focuses on the cognitive processes that allow self-generated actions to flow an overall analysis or plan for goal-directed problem-solving.

Complementing the RP results, motor β-power (13–30 Hz) desynchronization was deeper and more sustained for self-generated actions, particularly at sequence onset, while externally cued moves—as well as the second move of two-move self-generated problems—showed transient rebounds, reflecting reduced preparatory suppression. Although the relationship between the RP and motor β-band is still unclear, research suggests these two signals may operate independently. For example, recent findings indicate that the subjective feeling of readiness correlates with β-band amplitude but not with the RP, suggesting that β-power may be more closely tied to the conscious experience of preparing to act, whereas the RP might reflect unconscious preparatory processes (Parés-Pujolràs et al., 2023). Our results support the view that compound contralateral motor beta power may reflect strategic preparatory setting of a threshold for action under voluntarily control (Steinemann et al., 2018; He et al., 2020) to either facilitate or hinder action initiation (Kelly et al., 2021).

MVPA further strengthened the differences between self-generated and stimulus-driven actions, with decoding accuracy reliably distinguishing self-generated from stimulus-driven actions well before execution. Notably, classification performance was strongest for the first move of a sequence, with reduced differentiation at later stages, in parallel with the position-dependent effects observed in the RP and β-band results. However, after controlling for RT, the position gradient remained significant only for two-move sequences, suggesting that the first-move advantage in four-move sequences is partly related to longer planning times rather than purely condition-specific neural preparation. Despite this, robust above-chance decoding across all conditions—both before and after RT residualization—indicates that the preparatory neural signature distinguishing self-generated from stimulus-driven actions is a genuine and distributed feature of premovement EEG, not reducible to RT differences alone.

That is, the distinctive neural activities that characterize self-generated action are also linked to planning complex sequences of goal-directed actions. In this sense, the traditional neurophysiological concept of internally generated action can be linked to the psychological construct of problem-solving.

Our work therefore sheds light on the necessary and tight linkage between problem-solving and self-generated action. A key concept for this linkage is the cognitive process of planning. This concept has not been clearly operationalized in the previous cognitive neuroscience of self-generated actions, because of the focus on individual, often simple, and often arbitrary actions. For instance, failures of complex planning seen in frontal lobe patients are traditionally thought to reflect disruptions in the ability to plan extended chains of actions (Shallice, 1982). To date, research on self-generated action and on problem-solving have remained largely compartmentalized—with one tradition focusing on arbitrary choices made in the absence of clear goals and the other treating action as the trivial endpoint of antecedent complex cognition. Our findings challenge this divide. By embedding self-generated actions within structured, goal-directed problem-solving sequences, we show that the classical neural markers of self-generated actions—the RP and beta-band suppression—are not only present but systematically modulated by the planning demands of the task. These neural signatures were strongest when participants generated the first step of a novel problem, suggesting that self-generated actions are most engaged at the interface between cognition and action—when plans must be converted into self-initiated behavior.

Importantly, we demonstrate that preparatory signals are not merely markers of “free choice” but vary systematically with position within an action sequence and the associated planning demands.

Precursor neural markers of self-generated actions have previously been analyzed as potential correlates of decision (Libet et al., 1983) or commitment to move (Schultze-Kraft et al., 2016). By situating self-generated action within the broader cognitive architecture of goal pursuit, our neurocognitive study opens a new direction for research on self-generated actions. It invites a reconceptualization of self-generated actions not as isolated acts of will but as the key enabler of intelligent behavior.

## Synthesis

Reviewing Editor: Jennifer Dulin, Texas A…M University Decisions are customarily a result of the Reviewing Editor and the peer reviewers coming together and discussing their recommendations until a consensus is reached. When revisions are invited, a fact-based synthesis statement explaining their decision and outlining what is needed to prepare a revision will be listed below. The following reviewer(s) agreed to reveal their identity: NONE. Note: If this manuscript was transferred from JNeurosci and a decision was made to accept the manuscript without peer review, a synthesis may not be available.

Synthesis statement:

Both reviewers find the work timely and potentially impactful, highlighting the novel linkage between problem solving and voluntary action using high-temporal-resolution EEG, clear writing, and a generally strong experimental design. However, they raise substantive concerns about the conceptual framing (what counts as "volitional problem solving," adequacy of the instructed control, and title/terminology) and about the strength and interpretation of the neural analyses-especially RP characterization and baselining, the effect of action-locked baselines on decoding dynamics, and the divergence between ERP and decoding topographies. Reviewers also ask for stronger statistical support (e.g., 2×2 tests across sequence length and position), clearer evidence for complexity scaling (including separating end vs. intermediate moves), and targeted figure/visualization and reporting fixes. We are therefore issuing a revise-and-re-review decision to allow a thorough revision that addresses these conceptual, analytical, and presentation issues.

Reviewers' comments:

Reviewer #1:

In this manuscript, Seghezzi and Haggard investigate the neural mechanisms underlying what they term "volitional problem solving." Using EEG, they report that ERP amplitude, beta power, and decoding accuracy are stronger when participants actively solve a problem compared to when they simply follow instructed sequences. The research question is timely and interesting, and the manuscript is very clearly written with well-presented results. That said, some of the conclusions appear stronger than what the data directly support, and a few conceptual and analytical issues require clarification. Addressing the following points would considerably improve the manuscript.

1. The title raises questions about what counts as "volitional" versus "non-volitional" problem solving. The instructed condition does not actually involve problem solving at all, so it is unclear whether this is an appropriate control. The authors should justify why this comparison supports claims about "volitional problem solving," or else refine the title to something more precise, such as "Neural mechanisms of self-generated action sequences." More generally, the relationship between voluntary action and problem solving should be clarified as it is the main point of the manuscript. I am not disputing the fact that studying self-generated, goal-directed action sequence is very timely and important but some of the terminology in the manuscript, such as "volitional" needs clarification.

2. The manuscript describes "clear differences" between conditions and between sequence positions/lengths. However, these claims are not fully supported by the statistical analyses as presented. The presented results show that, for example, the ERP for self-generated action is different from instructed, but does not show any statistically significant "difference" between sequence positions/lengths. For example, a 2×2 ANOVA (sequence length vs. position) would provide a stronger test of main effects and interactions. This applies not only to ERP analyses but also to beta-band power and classifier performance.

3. The MVPA results show classifier performance declining as action onset approaches. The authors suggest this reflects a shift from planning to execution, but the decrease begins almost 500 ms before movement onset. This seems too early to attribute purely to motor execution. Could other explanations (e.g., variability in planning processes, overlap with non-motor signals) account for the pattern? Clarification is needed here.

4. The topographical distributions of decoding accuracy and ERP differences appear distinct (e.g., when comparing the first vs. later moves of 2-step sequences). Why do the authors think the neural sources highlighted by decoding diverge from those reflected in ERP contrasts? Some discussion of this discrepancy would strengthen the interpretation of the findings.

This is a well-written and potentially impactful paper. However, the conceptual framing around "volitional problem solving" and the strength of the statistical evidence both need refinement. Addressing the points above would help ensure that the claims are fully supported by the data.

Reviewer #2:

The neural correlates of voluntary action planning have often been studied in the context of spontaneous actions, whilst problem-solving research has focused on higher cognitive and representational aspects rather than action-planning. Here the authors bring these fields together to ask whether classic markers of voluntary action planning are mediated by whether the planned actions relate to goal-directed problem-solving. To do so they use an established problem-solving task that involves pre-planning a multi-step solution before acting. Importantly they used also deployed a control variant of this task, that did not require the pre-planning of multiple action steps. This enabled the authors to disentangle voluntary goal-directed actions that involved 'self-generated' multi-step action-planning from simple 'stimulus-driven' instructed actions. Using EEG they found differences between self-generated and stimulus-driven action conditions in the 1s prior to the action, for traditional markers of voluntary action planning i.e. differences in ERP amplitudes over central electrodes prior to the action, otherwise known as the readiness potential (RP), lower beta-band power, as well as stronger decoding of self-generated action plans than stimulus-driven ones.

Understanding the neural basis of more behavioural relevant voluntary actions would certainly be of great interest to many in the field, and I believe the experimental design is well-suited to address such questions. However, I have concerns about the interpretation of the neural analysis, especially with respect to the baseline timing, which I hope can benefit from clarification from the authors. I additionally have some minor concerns about the visualisation of the data, all of which I detail below -

Major

1. The interpretation of RP results

In figure 2, the authors show a difference in the amplitudes of the ERP over central electrodes between self-generated and stimulus-driven condition, especially for the 1st move in a sequence. Importantly, they baseline this activity with respect to a window around the action onset. Like this, they observe more negative ERPs in the self-generated than stimulus-driven conditions, which they suggest reflects a stronger RP when actions involve problem-solving vs. instruction following. However, the RP is typically characterised as a more 'negative-going' ERP (e.g. Schurger et al., 2021, Trends in Cognitive Science), a term which the authors also use repeatedly, implying a negative difference in the amplitude between the start of the measurement of the RP and time of action. In this sense, the stimulus-driven RP would appear to be stronger, as the difference between the amplitude at the time of the action (i.e. the baseline period) and 1s before, is significantly greater than that for the self-generated condition. It would be useful if the authors could clarify this point in case I have misunderstood how the RP is normally characterised. In addition, it would be useful if the authors could also plot the ERPs with respect to a pre-stimulus baseline, as this may illuminate the condition difference further, and is more comparable with previous findings.

2. Interpretation of decoding dynamics

For all panels in figure 4A, the authors suggest that the decoding accuracy of the condition (self-generated vs. stimulus-driven) decreases closer to the time of the action, and state that 'this decline presumably corresponds to a shift from premotor to primary motor cortical processing'. However, this can at least partially be explained by use of data baselined to the time around the action, as accuracy would necessarily be at chance at this time point.

3. Scaling with complexity

The authors argue that 'enhancement [of classic neural precursors of voluntary action] is proportional to the complexity of the problem' i.e. 4 vs. 2-move problems, based on significant differences between self-generated and stimulus-driven tasks for 4-move trials on 'other-moves' compared to insignificant differences for 'other-moves' on 2-move trials, for ERPs and beta-power, which they state are 'systematically modulated by the complexity'. They later point out that one explanation for the lack of difference between self-generated and stimulus driven ERPs/beta-power in the 2-mode task could relate to the presence of the end goal on the screen. This means that the end move of the self-generated could be solved in a similar way to the stimulus-driven case, i.e. by a visual match. If this is truly the case, this confound would also be likely to apply to the end-move of the 4-move task, in which case we would see similarly small (n.s.) differences between the self-generated and stimulus-driven conditions for the end move. It would therefore be informative if the authors would separate out the ERPs and beta-power results for 'final-move' from 'intermediate moves', to see whether this is the case. Furthermore, in light of this potential end-move confound and without an intermediate move for 2-move trials, it is difficult to say whether any conclusions about the impact of task complexity can be made. Nonetheless, it would be interesting to see the dynamics of the RP and beta power as a function of move number in the 4-move case to see whether there is a gradual decrease in differences, or only between the first 3 moves and the end-move. Finally, for the ERPs it appears as though the condition amplitude differences for the 1st move may be smaller for 4-move vs. 2-move trials, which would not fit with their argument. Perhaps the authors could test whether this is the case to clarify this point.

Minor -

- It may be worth excluding the topographic maps shown in figure 2a, as the authors are only interested in explaining the effects related to the RP and not other significant effects (e.g. the positive deflections). Thus, focusing their analysis from the beginning on their regions of interest would make more sense. Otherwise greater detail might be provided about any other effects observed.

- With respect to the behavioural results, more details on the statistical tests used could be provided e.g. in the text state that linear-mixed effects models were used with post-hoc tests. In the methods, provide more details on the post-hoc tests used.

- For figure 1c, the use of red and blue for 1st move and other move respectively, is misleading given these colours are later used to denote self-generated and stimulus driven. Changing the colours to denote the action condition rather than the move # would help prevent misunderstandings. Other changes that would allow easier direct comparison of the RT between different conditions e.g. shortening the axis for stim-driven, or overlaying the distributions, might also improve readability.

- For figure 2b, the legend is in an unfortunate position, which obscures the view of the stimulus-driven ERP dynamics. This should be moved elsewhere.

- In figure 4B, the authors depict channels where decoding of action-planning condition was above chance. However the use of a red-blue colour bar for topoplots is typically associated with 'positive' and 'negative' deflections around 0 (as in e.g. figure 2), and is potentially misleading to the reader, unless 0.025 represents a significant changepoint. Changing the limits from white to blue would make for a more intuitive graphic.

Typos

- Shorthands are often spelled out with brackets in the discussion e.g. readiness potential (RP), but not earlier on in the paper. These should be clear in the introduction for better understanding.

- In figure 1a, the 2nd move incorrectly has both the select and release parts labelled as '1st move'

- In figure 1b, the '+' between self-generated and the start configuration appears to serve no purpose.

- In figure 1c, reaction time is stated as being in 'ms' but this seems unlikely given the tiny values

- In the legend for figure 1c, the authors make reference to 'larger central dots' which indicate group mean, but the size does not seem to vary, rather the shape (diamond vs. circle)

## Author Response

Response to Reviewers eNeuro eN-TNWR-0316-25X We thank both reviewers for their careful reading and constructive feedback. We have addressed all comments in full. The main changes to the manuscript are as follows: (1) the RP and MVPA analyses are now presented without baseline correction; (2) factorial 2 × 2 cluster-based permutation tests have been implemented for all three EEG measures; (3) a move-position analysis separating final from intermediate moves has been added for both RP and β-band power; and (4) the original interpretation that preparatory neural activity scales with problem complexity has been revised to a position-based account centred on sequence stage and associated planning demands. Detailed responses to each comment are provided below.

Reviewer 1 Comment 1: Terminology and conceptual framing The title raises questions about what counts as "volitional" versus "non-volitional" problem solving. The instructed condition does not actually involve problem solving at all, so it is unclear whether this is an appropriate control. The authors should justify why this comparison supports claims about "volitional problem solving," or else refine the title to something more precise, such as "Neural mechanisms of self-generated action sequences." More generally, the relationship between voluntary action and problem solving should be clarified as it is the main point of the manuscript.

Response: We thank the reviewer for this important conceptual point.

We have therefore revised the title to "Neural mechanisms of self-generated action sequences in goal-directed problem solving" and modified the Introduction accordingly to clarify the relationship between self-generated action and problem solving in our paradigm. Specifically, we now explain that problem solving in this task requires participants to generate a sequence of actions that achieves a defined goal. The self-generated condition operationalises this requirement by asking participants to plan and execute the sequence without external instruction.

By contrast, the instructed condition controls for the motor and sensory demands of sequence execution while removing the need for action planning. The contrast between conditions therefore isolates the neural signature of internally generated action planning within a goal-directed problem-solving context, which we argue is the central construct captured by the task. We have added a paragraph to the Introduction (following the description of the two conditions) to make this distinction explicit.

In line with this clarification, we have replaced the term "volitional problem solving" with "planning goal-directed voluntary action sequences" throughout the relevant sections of the manuscript.

Comment 2: Statistical support for complexity scaling - factorial analysis The manuscript describes "clear differences" between conditions and between sequence positions/lengths. However, these claims are not fully supported by the statistical analyses as presented. A 2×2 ANOVA (sequence length vs. position) would provide a stronger test of main effects and interactions. This applies not only to ERP analyses but also to beta-band power and classifier performance.

Response: We thank the reviewer for this important methodological suggestion. We fully agree that the original analyses, which tested self-generated vs. stimulus-driven differences separately within each condition, did not directly assess whether these effects varied as a function of sequence position or sequence length. We have now implemented 2 × 2 factorial cluster-based permutation tests (sequence position × sequence length) for all three EEG measures (RP, β-band power, and MVPA decoding). Results are summarised below and reported in full in the revised manuscript.

Across all three measures, the factorial analyses revealed a consistent main effect of sequence position, with stronger self-generated versus stimulus-driven differences at the first move compared to subsequent moves. For the RP, this effect was robust and extended across the entire pre-movement window, confirming that the preparatory advantage is strongest at sequence initiation and diminishes thereafter. Crucially, there was no main effect of sequence length and no interaction, indicating that the position effect was comparable across 2-move and 4-move sequences.

A similar pattern was observed for β-band power. The factorial analysis again revealed a significant main effect of sequence position, with greater β suppression for self-generated relative to stimulus-driven actions at first moves compared to later moves. No main effect of sequence length was found. A post-movement interaction was observed, reflecting differences in β rebound dynamics between completed and ongoing sequences, but this did not alter the pre-movement position effect.

Finally, MVPA decoding showed converging evidence. Decoding accuracy was significantly higher for first moves than for subsequent moves, again yielding a main effect of sequence position without a main effect of sequence length. A modest interaction indicated that the first-move advantage was attenuated in longer sequences. We speculate that planning is done solely in advance for shorter sequences, while sequences of 4 moves and beyond may need some further ongoing planning 'on the fly' as the sequence unfolds. Planning capacity is clearly limited, so advance planning cannot be complete for long sequences.

Taken together, these new factorial analyses directly support our original claims. They demonstrate that the preparatory advantage for self-generated actions scales with sequence position rather than sequence length, and that this effect is consistent across univariate (RP, β) and multivariate (MVPA) measures.

Comment 3: MVPA decline near action onset The MVPA results show classifier performance declining as action onset approaches. The authors suggest this reflects a shift from premotor to primary motor cortical processing, but the decrease begins almost 500 ms before movement onset. Could other explanations account for the pattern? Response: We thank the reviewer for this insightful comment. Upon re-examination of the analysis, and following the advice of the second referee, we identified that the apparent decline in decoding accuracy prior to movement onset was driven by the pre-stimulus baseline correction applied during preprocessing.

Specifically, baseline subtraction introduced a systematic distortion in the late pre-movement period, which artificially reduced classifier performance as action onset approached. To address this, we reran the MVPA analysis on data preprocessed without baseline correction (0.05 Hz high-pass filter; no baseline subtraction).

In the revised analysis, the decline in decoding accuracy was no longer observed. Instead, decoding performance remained consistently and significantly above chance throughout the entire preparatory window (−1000 to 0 ms) across all four conditions (cluster-based permutation tests, all p < .001).

We have therefore replaced the original baseline-corrected MVPA results with these no-baseline results in the revised manuscript. The updated findings indicate that the neural signal distinguishing self-generated from stimulus-driven actions is sustained across the full preparatory period, without evidence for a late collapse in discriminability.

Accordingly, we have removed the previous interpretation suggesting a shift from premotor to primary motor cortical processing, as this was based on an artefactual pattern introduced by baseline correction. The revised results provide a more accurate characterisation of the data, showing stable and persistent condition-specific information up to movement onset.

Comment 4: Divergence between ERP and MVPA topographies The topographical distributions of decoding accuracy and ERP differences appear distinct. Why do the authors think the neural sources highlighted by decoding diverge from those reflected in ERP contrasts? Response: We thank the reviewer for raising this important methodological point.

In line with a related suggestion from Reviewer 2, we have removed the RP topographical plots from the revised manuscript to improve clarity and avoid potential overinterpretation of scalp distributions. We also note that our primary ERP and β analyses focus on a priori regions of interest, which provide a more direct and interpretable test of our hypotheses. For similar reasons, we have simplified the presentation of MVPA results to focus on decoding performance over time, rather than emphasising scalp topographies.

Conceptually, however, the divergence between ERP and MVPA topographies is expected and reflects fundamental differences between univariate and multivariate approaches. ERP contrasts capture differences in mean amplitude at each electrode, averaged across trials, and are therefore dominated by large, spatially coherent neural sources that produce strong dipolar scalp fields, such as the frontocentral negativity associated with the readiness potential. By contrast, MVPA identifies distributed spatial patterns of activity that reliably covary with condition, without requiring consistent mean differences at individual electrodes. As a result, brain regions can contribute to decoding even if they do not show significant univariate effects. This difference in sensitivity means that MVPA topographies need not, and often do not, match ERP difference maps.

However, we have removed the scalp topographies to avoid overinterpretation of classifier weight distributions, which do not provide a straightforward mapping onto underlying neural sources. Instead, we focus on time-resolved decoding accuracy, which directly addresses the question of whether condition-specific information is present.

Reviewer 2 Major Comment 1: Interpretation of RP results and baseline choice In figure 2, the authors show a difference in the amplitudes of the ERP over central electrodes between self-generated and stimulus-driven conditions, especially for the 1st move in a sequence. Importantly, they baseline this activity with respect to a window around the action onset. The RP is typically characterised as a more 'negative-going' ERP, implying a negative difference between the start of measurement and the time of action. In this sense, the stimulus-driven RP would appear to be stronger. It would be useful if the authors could clarify this point, and also plot the ERPs with respect to a pre-stimulus baseline.

Response: We thank the reviewer for this important point regarding the interpretation of the RP and the role of baseline correction. We agree that baseline choice can influence the apparent polarity and interpretation of readiness-potential differences, and we have revised our analyses accordingly.

A conventional pre-stimulus baseline is not straightforwardly interpretable in our paradigm, particularly for first moves. In the self-generated condition, participants engage in extended planning prior to initiating the first action, resulting in substantially longer and more variable preparation periods than in the stimulus-driven condition. As a result, any pre-stimulus interval is likely to contain condition-specific preparatory activity and cannot be assumed to reflect a common neutral baseline across conditions. Baseline-correcting to such a window would therefore risk distorting the true magnitude and temporal profile of preparatory signals. As in many self-generated action paradigms, there can be no 'natural' baseline, akin to the baseline prior to stimulus presentation for an evoked potential, because it is unknown when preparation begins, so the natural option of setting the baseline just prior to this point does not work.

In the original submission, both the RP and MVPA analyses were performed with baseline correction centred on the time of the action (−5 to +5 ms). Following the reviewer's comment, we re-evaluated this preprocessing step. In particular, we found that baseline subtraction introduced interpretational ambiguities in the MVPA analysis, where it produced an artifactual reduction in decoding accuracy in the late pre-movement period. We therefore re-ran all analyses without baseline correction (Schurger, 2018).

In the revised manuscript, we consistently report results from data processed without baseline correction across all measures (RP, β-band power, and MVPA). This ensures a coherent analytical approach and avoids introducing measure-specific preprocessing assumptions. In addition, the resulting RP waveforms follow the conventional gradual build-up of premovement negativity, facilitating comparison with the existing literature.

By analysing the data without baseline correction, we avoid imposing assumptions about when preparatory activity begins and instead characterise the full temporal evolution of the signal. Importantly, the revised analyses yield a clear and consistent pattern across all three measures, with sustained differences between self-generated and stimulus-driven conditions throughout the preparatory window.

In response to the reviewer's suggestion, we have clarified this issue in the manuscript and explicitly discuss how baseline choice affects RP interpretation. We have also updated the relevant figures and captions to reflect the revised analysis.

Schurger, A. (2018). Specific Relationship between the Shape of the Readiness Potential, Subjective Decision Time, and Waiting Time Predicted by an Accumulator Model with Temporally Autocorrelated Input Noise. eNeuro, 5(1). https://doi.org/10.1523/ENEURO.0302-17.2018 Major Comment 2: MVPA decline near action onset For all panels in figure 4A, the authors suggest that the decoding accuracy of the condition decreases closer to the time of the action, and state that this decline presumably corresponds to a shift from premotor to primary motor cortical processing. However, this can at least partially be explained by use of data baselined to the time around the action, as accuracy would necessarily be at chance at this time point.

Response: We thank the reviewer for this important observation: we should have seen this artefact, and now we have learned our lesson! We agree completely. Upon re-examination, we found that the apparent decline in decoding accuracy prior to action onset in the original analysis was artefactually produced by baseline correction centred on the time of action. As the reviewer notes, this procedure necessarily forces decoding accuracy towards chance at the baseline time point.

When the analysis was rerun on data preprocessed without baseline correction (0.05 Hz high-pass filter; no baseline subtraction), this decline was no longer observed. Instead, decoding accuracy remained consistently and significantly above chance throughout the entire preparatory window (−1000 to 0 ms) in all four conditions (cluster-based permutation tests, all p < .001).

We have therefore replaced the original baseline-corrected MVPA results with the no-baseline analysis in the revised manuscript. The updated results provide a clearer and more accurate characterisation of the data, showing that condition-specific information is sustained throughout the preparatory period rather than decreasing prior to movement onset.

Accordingly, we have removed the previous interpretation suggesting a shift from premotor to primary motor cortical processing, as this was based on an artefactual pattern introduced by baseline correction. The revised manuscript has been updated to reflect this change in both the Results and Discussion.

Major Comment 3: Scaling with complexity - move-position analysis The authors argue that enhancement is proportional to the complexity of the problem based on significant differences for 4-move trials on other-moves compared to insignificant differences for other-moves on 2-move trials. It would be informative if the authors would separate out the ERPs and beta-power results for final-move from intermediate moves, to see whether the end-goal confound applies. Furthermore, it would be interesting to see the dynamics of the RP and beta power as a function of move number in the 4-move case.

Response: We thank the reviewer for this important observation. Following this suggestion, we re-analysed both the RP and β-band data at the level of individual move positions, explicitly separating final moves from intermediate moves across both sequence types.

For the RP, this analysis revealed a clear dissociation between non-final and final moves. A robust self-generated preparatory advantage was observed at all non-final moves, including the first move of both 2- and 4-move sequences and the second move of 4-move sequences. In contrast, this effect was absent at the final move of both sequence types. In the 4-move condition, the magnitude of the RP difference showed a graded reduction across successive moves, which was confirmed by a significant linear trend from the first to the final move. These results indicate that the preparatory advantage is not simply driven by sequence length or overall complexity, but instead varies systematically with position in the sequence and the associated planning demands.

For β-band power, the pattern differed in an important respect. While β suppression also showed stronger self-generated effects at earlier moves, significant differences between self-generated and stimulus-driven conditions were present at all move positions, including the final move of both sequence types. However, the magnitude of this effect showed a numerical decrease across positions in the 4-move sequence, although this trend did not reach statistical significance. This suggests that, unlike the RP, β suppression reflects a more sustained state of motor engagement that persists even when forward planning demands are reduced.

Taken together, these analyses clarify that the apparent scaling with "complexity" in the original results is better explained by sequence position and the associated planning requirements. By separating final and intermediate moves, we show that the preparatory advantage for self-generated actions is strongest at early stages of the sequence and diminishes as the sequence progresses, rather than simply increasing with sequence length.

These results have now been incorporated into the revised manuscript, and the interpretation has been updated accordingly.

Major Comment 3b: First-move amplitude comparison between sequence types For the ERPs it appears as though the condition amplitude differences for the 1st move may be smaller for 4-move vs. 2-move trials, which would not fit with their argument. Perhaps the authors could test whether this is the case.

Response: We tested this directly by comparing the self-generated versus stimulus-driven difference at the first move between 2-move and 4-move sequences. This analysis revealed no significant difference between sequence lengths (no significant clusters), indicating that the magnitude of the preparatory effect at the first move was comparable across 2- and 4-move trials.

This result suggests that the apparent difference in amplitude observed in the figure is not in fact a statistically reliable effect. Importantly, it confirms that the preparatory advantage at the first move does not scale with sequence length, and therefore does not support a simple "greater complexity leads to greater amplitude" account.

We have added this comparison to the revised manuscript to clarify this point.

Minor Comments Topographic maps in Figure 2a It may be worth excluding the topographic maps shown in figure 2a, as the authors are only interested in explaining the effects related to the RP and not other significant effects (e.g. the positive deflections).

Response: We have removed the topographic maps from Figure 2a. This keeps the focus of the figure on the effects of theoretical interest and avoids inviting questions about positive deflections and other features for which we do not have a principled account.

Statistical reporting in behavioural results More details on the statistical tests used could be provided, e.g. in the text state that linear-mixed effects models were used with post-hoc tests. In the methods, provide more details on the post-hoc tests used.

Response: We thank the reviewer for this suggestion. We have revised the methods sections to provide greater detail on the statistical approach. We have expanded the description of the post-hoc tests to clarify that planned contrasts were conducted using estimated marginal means (emmeans package), and we now specify the nature of each contrast tested: (1) a difference-in-differences contrast testing whether the sequence position effect (first vs. other moves) differed between action types as a function of sequence length, and (2) contrasts testing whether the first-move slowing differed between 2-move and 4-move sequences separately for each action type. All post-hoc p-values were corrected using the Bonferroni method.

Figure 1c colour scheme The use of red and blue for 1st move and other move respectively, is misleading given these colours are later used to denote self-generated and stimulus driven. Changing the colours to denote the action condition rather than the move # would help prevent misunderstandings.

Response: We thank the reviewer for this helpful suggestion. We have revised Figure 1c so that colours consistently denote action condition (self-generated vs stimulus-driven), rather than move position.

Figure 2b legend position The legend is in an unfortunate position, which obscures the view of the stimulus-driven ERP dynamics. This should be moved elsewhere.

Response: The legend in Figure 2 has been moved to the left side of the figure.

Figure 4B colour scale In figure 4B, the use of a red-blue colour bar for topoplots is typically associated with positive and negative deflections around 0, and is potentially misleading. Changing the limits from white to blue would make for a more intuitive graphic.

Response: We thank the reviewer for this helpful suggestion. More generally, we decided to simplify the presentation of MVPA results to focus on time-resolved decoding accuracy, which directly addresses the presence of condition-specific information.

This change also avoids potential overinterpretation of spatial patterns derived from classifier outputs, which do not provide a straightforward mapping onto underlying neural sources. The revised figures therefore present decoding performance over time only.

Typographical corrections Shorthands are often spelled out with brackets in the discussion e.g. readiness potential (RP), but not earlier on in the paper. In figure 1a, the 2nd move incorrectly has both the select and release parts labelled as '1st move'. In figure 1b, the '+' between self-generated and the start configuration appears to serve no purpose. In figure 1c, reaction time is stated as being in 'ms' but this seems unlikely given the tiny values. In the legend for figure 1c, the authors make reference to 'larger central dots' which indicate group mean, but the size does not seem to vary, rather the shape.

Response: Response: Thank you for your careful attention in pointing these out: we apologise for these oversights, and , we have corrected all five these issues in the revised manuscript.
